# The role of Psl in the failure to eradicate *Pseudomonas aeruginosa* biofilms in children with cystic fibrosis

**DOI:** 10.1038/s41522-021-00234-3

**Published:** 2021-08-04

**Authors:** Amanda J. Morris, Lindsay Jackson, Yvonne CW Yau, Courtney Reichhardt, Trevor Beaudoin, Stephanie Uwumarenogie, Kevin M. Guttman, P. Lynne Howell, Matthew R. Parsek, Lucas R. Hoffman, Dao Nguyen, Antonio DiGiandomenico, David S. Guttman, Daniel J. Wozniak, Valerie J. Waters

**Affiliations:** 1 Translational Medicine, Research Institute, Hospital for Sick Children, Toronto, ON Canada; 2 Division of Microbiology, Department of Pediatric Laboratory Medicine, The Hospital for Sick Children, University of Toronto, Toronto, ON Canada; 3 Department of Microbiology, University of Washington, Seattle, WA USA; 4 Program in Molecular Medicine, Research Institute, The Hospital for Sick Children, Toronto, ON Canada; 5 Department of Biochemistry, University of Toronto, Toronto, ON Canada; 6 Departments of Pediatrics and Microbiology, University of Washington, Seattle, WA USA; 7 Department of Medicine, McGill University, Montreal, QC Canada; 8 Discovery Microbiome, Microbial Sciences, BioPharmaceuticals R&D, AstraZeneca, Gaithersburg, USA; 9 Department of Cell and Systems Biology, University of Toronto, Toronto, ON Canada; 10 Departments of Microbial Infection and Immunity, Microbiology, Ohio State University, Columbus, OH USA; 11 Division of Infectious Diseases, Department of Pediatrics, The Hospital for Sick Children, Toronto, Canada; 12 Department of Pediatrics, University of Toronto, Toronto, ON Canada

**Keywords:** Biofilms, Clinical microbiology

## Abstract

The exopolysaccharide Psl contributes to biofilm structure and antibiotic tolerance and may play a role in the failure to eradicate *Pseudomonas aeruginosa* from cystic fibrosis (CF) airways. The study objective was to determine whether there were any differences in Psl in *P. aeruginosa* isolates that were successfully eradicated compared to those that persisted, despite inhaled tobramycin treatment, in children with CF. Initial *P. aeruginosa* isolates were collected from children with CF undergoing eradication treatment, grown as biofilms and labeled with 3 anti-Psl monoclonal antibodies (Cam003/Psl0096, WapR001, WapR016) before confocal microscopy visualization. When grown as biofilms, *P. aeruginosa* isolates from children who failed antibiotic eradication therapy, had significantly increased Psl0096 binding compared to isolates from those who cleared *P. aeruginosa*. This was confirmed in *P. aeruginosa* isolates from the SickKids Eradication Cohort as well as the Early Pseudomonas Infection Control (EPIC) trial. Increased anti-Psl antibody binding was associated with bacterial aggregation and tobramycin tolerance. The biofilm matrix represents a potential therapeutic target to improve *P. aeruginosa* eradication treatment.

## Introduction

Cystic fibrosis (CF) is a genetic disease characterized by *Pseudomonas aeruginosa* pulmonary infection^1^. To prevent the detrimental outcomes associated with chronic infection, antimicrobial treatment is used to eradicate initial *P. aeruginosa* infection^2–5^. However, in 10–40% of cases, eradication therapy fails, with no clear superiority of one antibiotic regimen over another, and the reasons for this are not entirely understood^6^.

In addition to antibiotic treatment, clearance of the organism from the airways depends on mucociliary action and immune-mediated mechanisms such as phagocytosis by neutrophils^7,8^. Studies that have examined outcomes of eradication therapy have not identified any host factors, such as gender or lung function, that are associated with failure to clear *P. aeruginosa*^9,10^. *P. aeruginosa* phenotypes characteristic of chronic pulmonary infection, such as mucoidy status, decreased motility and wrinkly colony morphology, have occasionally been identified as risk factors for failure of antibiotic eradication therapy^11,12^.

Using a collection of new onset *P. aeruginosa* isolates from children with CF undergoing antibiotic eradication treatment, we showed that Staphylococcal protein A (SpA) bound to the exopolysaccharide Psl in *P. aeruginosa* isolates that failed eradication therapy but bound much less in isolates successfully cleared^13^. This Psl-SpA interaction led to *P. aeruginosa* aggregation within biofilms and tolerance to high concentrations of tobramycin. These data suggest that, although the reasons for the failure of eradication therapy are likely multifactorial, Psl may be playing a role. Psl is a neutral repeating pentasaccharide that contributes to cell–cell and cell–substrate attachment adhesion, aggregation and biofilm formation in vitro^14–19^. Patients with invasive *P. aeruginosa* infections have serum antibodies against Psl, however, we do not know whether this occurs in CF patients^20^. Psl also protects *P. aeruginosa* from antimicrobials, including tobramycin and ciprofloxacin^21^, by forming a barrier matrix, and from activities of the innate immune system such as phagocytosis by neutrophils^22–24^. However, its contribution to the persistence of *P. aeruginosa* in the CF airways following inhaled antibiotic treatment is not known.

Therefore, the goals of this study were to examine Psl production and function in *P. aeruginosa* isolates that were successfully eradicated compared to those that persisted, despite inhaled tobramycin treatment, in the airways of children with CF. To do so, we used two sets of *P. aeruginosa* isolates, from the SickKids Eradication Cohort and the Early Pseudomonas Infection Control (EPIC) trial^11,25^. In addition, we used three separate anti-Psl antibodies (Cam003/Psl0096, WapR001 and WapR016), which recognize distinct epitopes and vary in their characteristics for promoting opsonization and phagocytosis and preventing epithelial cell binding^20,26^. We identified differences in Psl0096 binding between persistent and eradicated *P. aeruginosa* isolates with corresponding differences in bacterial aggregation and tobramycin tolerance.

## Results

### Quantification of Psl production in eradicated and persistent *P. aeruginosa* isolates

**Fig. 1:**
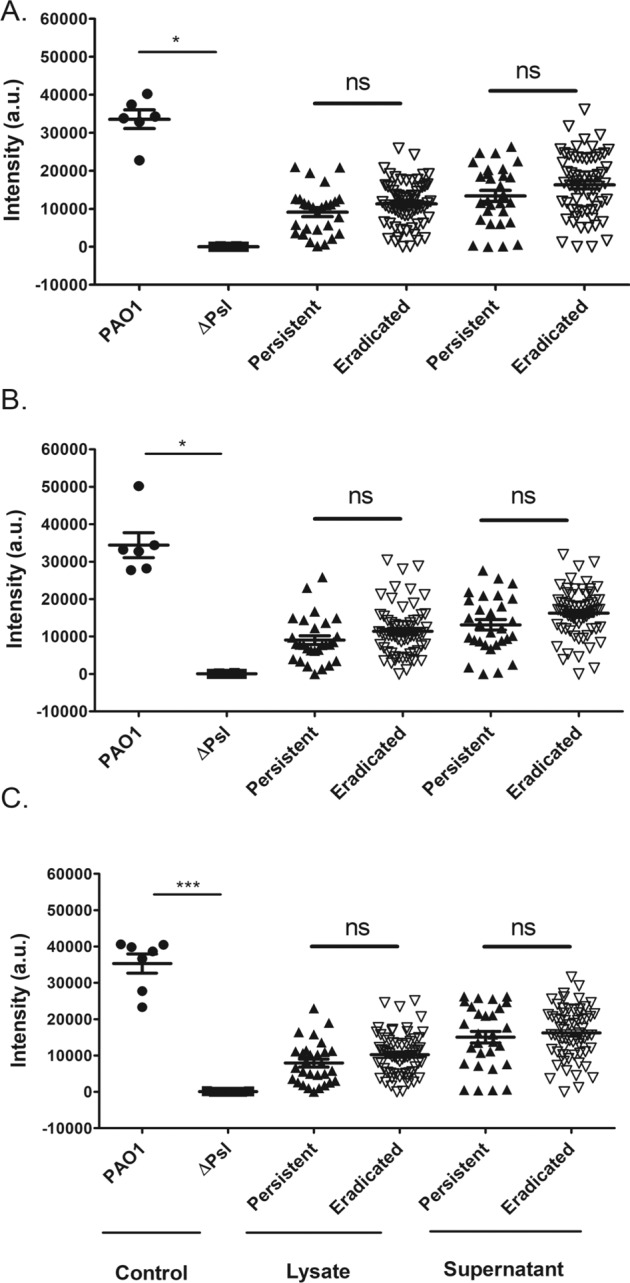
Dot blot analysis of Psl levels in persistent (*N* = 29, black triangles) versus eradicated (*N* = 63, white triangles) *P.aeruginosa* isolates from SickKids cohort. SickKids *P. aeruginosa* isolates, PAO1 and ΔPsl (Psl deficient *P. aeruginosa*) were grown as biofilms. The supernatant and lysate were spotted on nitrocellulose membrane and incubated with Cam003 (**A**), WapR001 (**B**) or WapR016 (**C**) anti-Psl antibodies. Bound antibody was detected with a chemiluminescent substrate after secondary antibody incubation, and signal intensities were analyzed using the ChemiDoc Imaging System. The mean intensity values for each isolate (done in triplicate) were plotted as arbitrary units (a.u.) with standard error of the mean (SEM). Statistics were performed using non-parametric Mann–Whitney test ***p < 0.001, *p < 0.05, ns not significant.

Initial investigations focused on determining whether there were any differences in the quantity of secreted and cell-associated Psl produced by eradicated and persistent isolates from the complete SickKids collection (29 persistent, 63 eradicated isolates). Biofilms were grown and sonicated to disrupt cell aggregation and lyse the bacteria. The supernatant and lysate were then incubated with three separate anti-Psl monoclonal antibodies recognizing distinct epitopes (Cam003/Psl0096, WapR001 and WapR016). Figure 1 illustrates that there was no difference in the amount of Psl detected via densitometric analysis of signal intensity between the eradicated (*N* = 63 isolates) and persistent (*N* = 29 isolates) isolates, either in the supernatant or lysate components.

### Comparative anti-Psl antibody binding to in vitro grown biofilms of eradicated and persistent isolates

In order to visualize Psl within intact biofilms (without sonication), *P. aeruginosa* were grown as biofilms and then stained with fluorescently labeled anti-Psl antibodies. For these detailed experiments, seven eradicated and seven persistent isolates from the SickKids collection were used. These isolates were chosen to represent 1 isolate per patient and to have similarities in other phenotypic characteristics which may influence eradication success, such as motility, mucoidy status, and planktonic tobramycin minimum inhibitory concentrations (MICs) between the eradicated and persistent groups of isolates, as previously published^13^.

**Fig. 2:**
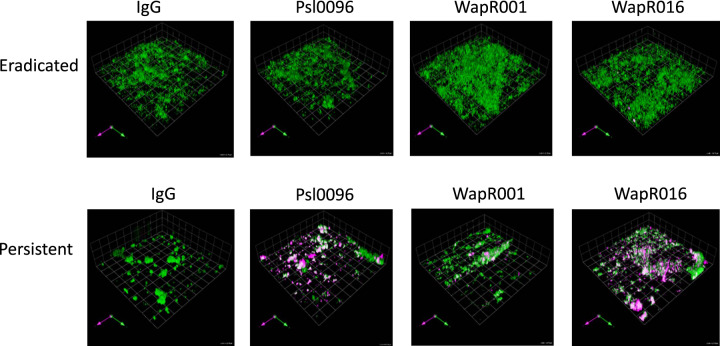
*P. aeruginosa* isolates were grown as biofilms for 48 h then incubated for 90 min with fluorescently labeled anti-Psl antibodies (IgG control, Psl0096, WapR01, and WapR016). Representative images are shown for a SickKids eradicated (Pa50) and persistent (Pa565) isolate, where biofilms are stained green and the antibody binding is magenta.

**Fig. 3:**
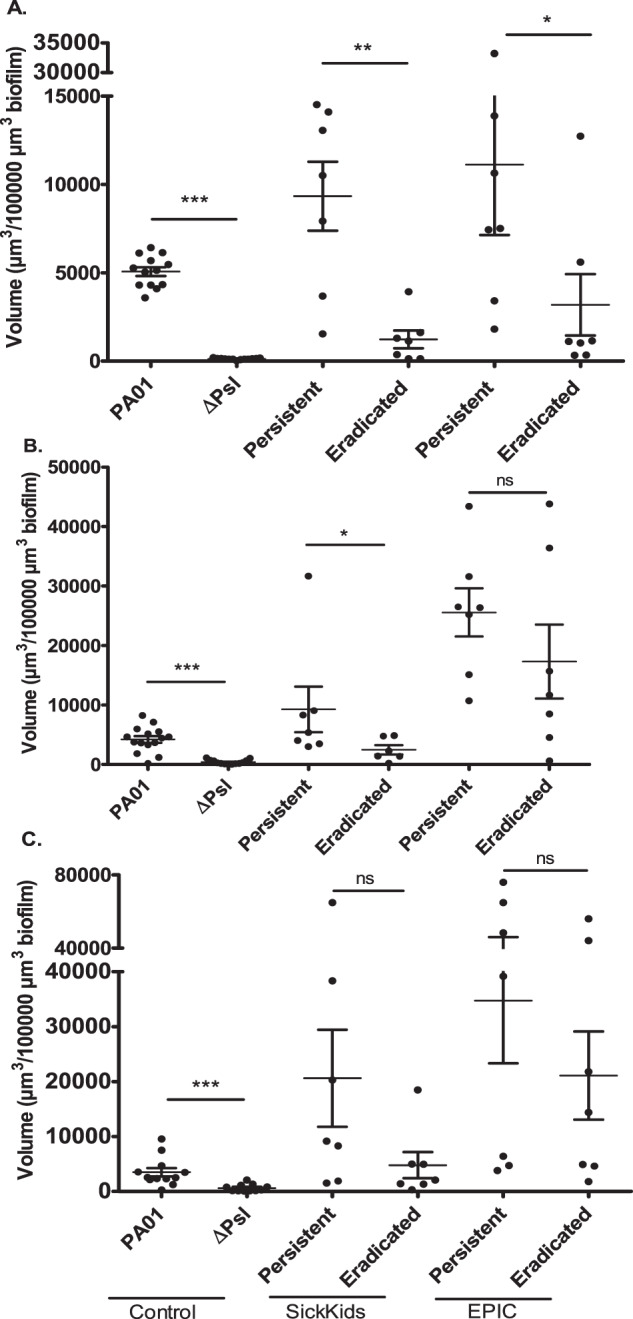
Anti-Psl antibody binding. SickKids *P. aeruginosa* isolates (*N* = 7 Persistent; *N* = 7 eradicated), EPIC *P. aeruginosa* isolates (*N* = 7 Persistent; *N* = 7 eradicated), laboratory *P. aeruginosa* strain PAO1 and *Δpsl* (Psl deficient PAO1) were grown as biofilms for 48 h then incubated for 90 min with fluorescently labeled monoclonal anti-Psl antibodies, **A** Psl0096, **B** WapR001, and **C** WapR016. The mean antibody fluorescence volume (µm^3^/100,000 µm^3^ biofilm) for each isolate (done in triplicate) was calculated and the mean of all isolates was plotted with standard error of the mean (SEM). Statistics were performed using non-parametric Mann–Whitney test ***p < 0.001, **p < 0.01, *p < 0.05, ns not significant.

Representative images of an eradicated and a persistent isolate are shown in Fig. 2, demonstrating increased anti-Psl antibody binding in the persistent *P. aeruginosa* biofilm. Figure 3 depicts the volume of anti-Psl antibody staining (per 100,000 µm^3^ of biofilm) in all persistent versus eradicated isolates. In the SickKids collection, there was significantly more anti-Psl antibody binding of Psl0096 (Fig. 3A) and WapR001 (Fig. 3B) antibodies in persistent compared to eradicated *P. aeruginosa*.

To validate these findings in a second dataset, we repeated the same experiments using seven persistent and seven eradicated isolates (from separate patients) from the EPIC cohort. Although there were no differences between groups using the WapR001 and WapR016 antibodies, Psl0096 bound significantly more in the persistent *P. aeruginosa* isolates compared to the eradicated ones, as seen in the SickKids cohort (Fig. 3).

### Effects of anti-Psl antibody binding in the presence of tobramycin in *P. aeruginosa* biofilms

**Fig. 4:**
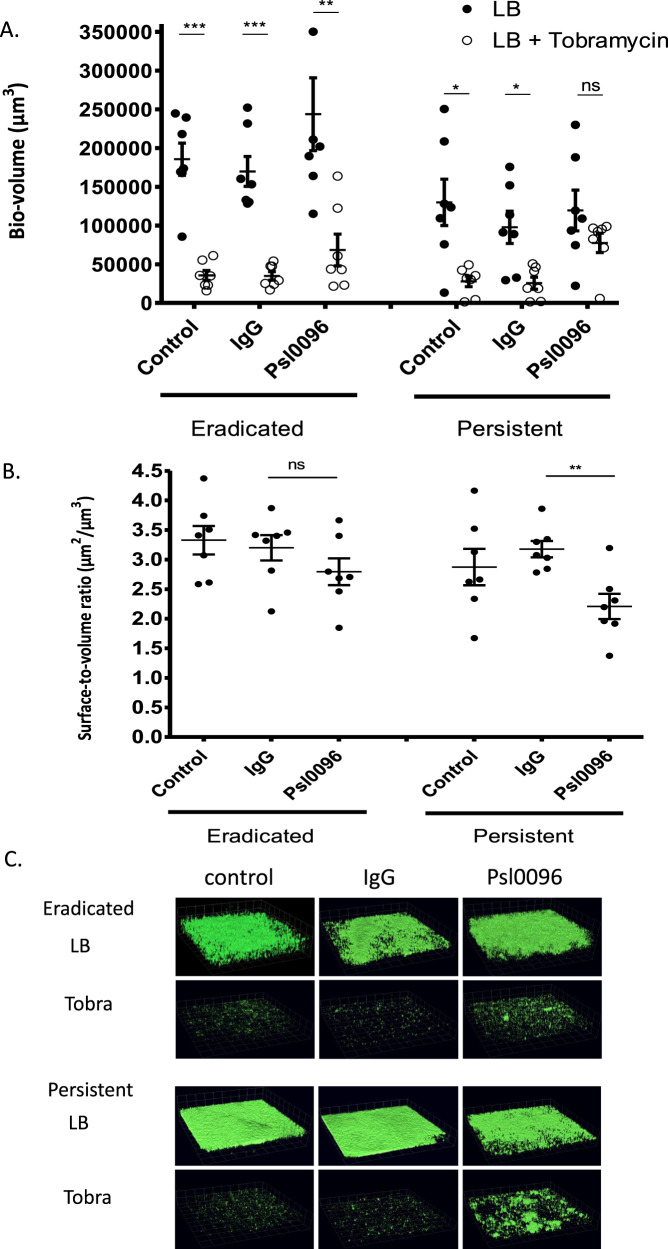
SickKids *P. aeruginosa* isolates (*N* = 7 persistent; *N* = 7 eradicated) were grown as biofilms for 24 h after which antibodies (LB control, IgG or anti-Psl 0096) and tobramycin 1000 µg/ml (or LB alone) were added for the following 24 h before imaging was performed. **A** The mean biofilm volume (measured in µm^3^) for each isolate (done in triplicate) was calculated and the mean of all isolates was plotted with standard error of the mean (SEM). Comparisons with and without tobramycin treatment were performed for each condition using non-parametric Mann–Whitney test ***p < 0.001, **p < 0.01, *p < 0.05, ns not significant. **B** In the presence of tobramycin, aggregation was measured using biofilm surface to volume ratio. The mean biofilm surface to volume ratio (µm^2^/µm^3^) was calculated for each isolate (done in triplicate) and the mean of all isolates was plotted with standard error of the mean (SEM). Comparisons to IgG control were performed for each condition using non-parametric Mann–Whitney test ***p < 0.001, **p < 0.01, *p < 0.05, ns: not significant. **C** Representative images are shown for a persistent (Pa375) and eradicated (Pa558) isolate.

We previously noted that Spa-Psl binding led to bacterial aggregation and tobramycin tolerance in persistent isolates^13^. To determine whether this finding was a Psl specific phenomenon and would occur with the increased binding of Psl0096 to persistent isolates, we investigated the effects of anti-Psl antibody binding on tobramycin resistance in *P. aeruginosa* biofilms. Biofilms of the SickKids seven eradicated and seven persistent isolates were grown for 24 h and then exposed to Psl0096 followed by tobramycin for an additional 24 h. Tobramycin significantly reduced the amount of biofilm volume for the eradicated isolates in media alone as well as in the presence of IgG and Psl0096 (Fig. 4A). However, for the persistent isolates, tobramycin significantly reduced biofilm volume only in the media and IgG control conditions; in the presence of Psl0096, there was no significant reduction in *P. aeruginosa* biovolume. Furthermore, in comparison to the IgG controls (to account for the non-specific effects of antibody binding), persistent isolates also had significant reductions in biofilm surface to volume ratio in the presence of Psl0096, indicating increased aggregation; increased aggregation was not observed in the eradicated group (Fig. 4B). Figure 4C shows representative images of a persistent and an eradicated isolate.

**Fig. 5:**
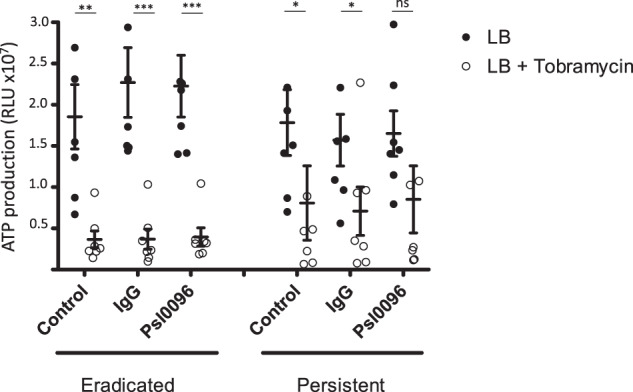
SickKids *P. aeruginosa* isolates (*N* = 7 persistent; *N* = 7 eradicated) were grown as biofilms for 24 h after which antibodies (LB control, IgG or anti-Psl 0096) and tobramycin (or LB alone) were added for the following 24 h before ATP was measured. The mean ATP activity (measured in Relative Light Units (RLU) ×10^7^) for each isolate (done in triplicate) was calculated and the mean of all isolates was plotted with standard error of the mean (SEM). Comparisons with and without tobramycin treatment were performed for each condition using non-parametric Mann–Whitney test ***p < 0.001, **p < 0.01, *p < 0.05, ns not significant.

We then used an ATP assay to investigate the metabolic activity of the remaining *P. aeruginosa* in the presence of tobramycin. For the eradicated isolates, tobramycin again significantly reduced ATP production under all conditions (Fig. 5). In the persistent group, tobramycin significantly reduced ATP production only in the control conditions and not in the presence of Psl0096. When colony-forming units (CFUs) were measured under the same conditions, however, tobramycin was able to significantly reduce the colony count in both the persistent and eradicated groups (Supplemental Fig. 1).

### Psl polymorphism among eradicated and persistent *P. aeruginosa* isolates

To determine whether there were any underlying genetic differences among the seven persistent and seven eradicated SickKids isolates, we examined the 14 locus Psl region from *pslA – pslN* and identified all non-synonymous (missense) mutations. These genes total to 17,657 bp and span the region from 2,453,667 to 2,472,076 on the PAO1 genome (note that some *psl* genes overlap). There was an average of 0.81 ± 0.549 (stddev) non-synonymous polymorphisms per strain across all 14 loci, with no variation found in *pslD*, and only one polymorphism found in both *pslA* and *pslG*. The most polymorphic locus was *pslB* with an average of 1.57 non-synonymous polymorphisms per strain. There were no statistically significant differences in the number of polymorphisms per gene (or when all genes were considered together) between eradicated and persistent strains (Supplemental Fig. 2).

### CdrA expression in eradicated and persistent *P. aeruginosa* isolates

Given that CdrA is a Psl associated matrix protein that plays a significant role in aggregation, we investigated the expression of CdrA in the 21 persistent and 25 eradicated SickKids isolates grown as biofilms. Supplemental Fig. 3 shows that CdrA expression was detected (particularly in the supernatant fraction) in a similar number of persistent compared to eradicated isolates. Size differences in CdrA were observed between isolates in the western blot analysis. This is consistent with past observations of *P. aeruginosa* isolates^27^.

## Discussion

This study demonstrated that there was increased binding of anti-Psl antibodies in *P. aeruginosa* isolates (when grown as biofilms) from CF patients, from both the SickKids and EPIC cohort, who failed antibiotic eradication therapy compared to those who did not; anti-Psl antibody binding led to the aggregate formation in biofilms and tobramycin tolerance.

To our knowledge, this is the largest study to examine the specific contribution of Psl in the failure to eradicate *P. aeruginosa* with antibiotic therapy in children with CF. Psl is involved in early bacterial surface attachment and is an important structural component of the extracellular matrix of biofilms^28,29^. Psl production can also confer resistance to antibiotics such as colistin, tobramycin, and ciprofloxacin, likely through a protective barrier effect^21^. Furthermore, Psl prevents complement deposition and opsonization, inhibiting bacterial killing by phagocytes^23,30^. In vitro studies of Psl have included *P. aeruginosa* strains from CF patients with chronic infection but not from those with early infection^31,32^. It is important to understand the significance of Psl in initial *P. aeruginosa* colonization which is more amenable to antibiotic treatment and clearance than chronic infection^6^.

In our study, we noted differential binding of anti-Psl antibodies to biofilm cells of eradicated versus persistent isolates. Monoclonal antibodies to the Class I epitope (Cam003 or its affinity optimized derivative, Psl0096) were previously found to be the most active in promoting opsonophagocytic killing, in preventing *P. aeruginosa* from binding to epithelial cells and preferentially bind to the surface of in vitro grown biofilms^20,26^. We also noted that mAb Psl0096 was the most discriminatory mAb in terms of binding between persistent and eradicated isolates, both for the SickKids and EPIC cohort. There were no significant differences in the number of polymorphisms in Psl genes between the groups to explain our finding. Furthermore, the amount of Psl as measured by dot blot analysis (using Psl0096) was similar between both groups. However, when *P. aeruginosa* was allowed to form a mature biofilm, undisturbed by sonication or processing, the increased affinity to Psl0096 was demonstrated. It is possible that persistent isolates grown as biofilms produce more surface Psl with a conformation that allows Psl0096 to bind more avidly than do eradicated isolates. In a screening study using synthetic oligosaccharides, Class I mAbs (Cam003/Psl0096) did not bind any of the oligosaccharides, suggesting the presence of a Psl-associated functional group or modification that is sensitive to experimental conditions such as alkaline exposure^33^.

We also observed that anti-Psl antibody binding was associated with aggregation, particularly in the persistent isolates. Earlier investigations actually noted a decrease in aggregation when PAO1 was exposed to Psl mAbs, however, such studies were done with planktonic growth of *P. aeruginosa* in culture medium^34^. The aggregation we noted in our study was Psl specific rather than simply an effect of adding antibodies as the aggregation seen with anti-Psl antibodies was significantly greater than that seen with equal concentrations of IgG. The bacterial aggregation may have been caused by the cross-linking of anti-Psl antibodies between *P. aeruginosa* cells. The mechanism did not appear to be due to an increase in the expression of CdrA, a Psl-associated matrix protein which enhances bacterial aggregation^35,36^, in persistent versus eradicated isolates. Bacterial aggregation is known to be a mechanism of antibiotic resistance;^37^ the aggregation noted in our persistent isolates was associated with tolerance (persistence under confocal imaging and greater metabolic activity as measured by ATP assay) to high levels of tobramycin, concentrations of 1000 µg/ml, achievable only with inhalation therapy^38^. There is also the possibility of non-Psl-mediated mechanism to account for the increased survival of the persistent isolates.

The presence of *P. aeruginosa* aggregates expressing Psl in the airways of a CF patient with chronic infection has been demonstrated by our collaborators in a study using immunohistochemical techniques to visualize CF sputum^39^. It is not known what the specific trigger for this aggregation may be in vivo; simply growing the persistent isolates as biofilms did not reveal persistence in the presence of tobramycin, suggesting that the ability to form biofilms alone may not fully explain persistence in the CF airways^40^. We previously showed that Staphylococcal protein A (SpA) can bind to Psl in our persistent *P. aeruginosa* strains leading to aggregation and tobramycin tolerance^13^. However, this aggregative phenotype is not dependent on SpA, as many of these CF patients who failed eradication therapy were not co-infected with *Staphylococcus aureus* and this aggregation phenotype can be replicated in the persistent isolates with the addition of anti-Psl antibodies. Patients with invasive *P. aeruginosa* infections are known to have serum antibodies against Psl, however, it is not known whether anti-Psl antibodies can be detected in the sputum of CF patients colonized with *P. aeruginosa*^20^. There are many factors that can contribute to the formation of bacterial aggregates in CF airways, which occur by two general mechanisms: bridging and depletion aggregation^41–45^. Host-derived polymers at the site of chronic infections may physically connect bacterial cells via a bridging mechanism leading to Psl mediated aggregation^39^.

This study had several limitations. The SickKids Eradication cohort was limited to children with CF who could produce sputum and the study defined eradication based on an early time point (1 week post treatment)^11,46^. To make our findings more generalizable, we validated our results using a second isolate collection, from the EPIC cohort, that was obtained from oropharyngeal swabs, and for which eradication was defined at the 3 month post treatment follow up visit^25^. Thus, our data suggest that differences in Psl are important in the persistence of *P. aeruginosa* infection in most children with CF. In addition, we do not have the data in either cohort to confirm that failure of eradication treatment was due to the persistence of the same strain of *P. aeruginosa*, as these were clinical isolates and genotyping is not done routinely in the clinical microbiology laboratory. We focused our study on the role of Psl solely within *P. aeruginosa* biofilms but have not included the potential contribution of host factors such as neutrophils, which clearly interact with Psl^34,47^. Finally, we visualized *P. aeruginosa* biofilms in vitro but have not yet confirmed our study findings in vivo, directly in the sputum of children with CF; this is the focus of a currently ongoing observational study.

In conclusion, we demonstrated that *P. aeruginosa* isolates that fail to be cleared from the airways of children with CF following inhaled tobramycin therapy, have increased binding to anti-Psl antibodies when grown as biofilms, leading to aggregation and tobramycin tolerance. These components of the biofilm matrix represent possible therapeutic targets to improve the success of early *P. aeruginosa* eradication treatment.

## Methods

### *P. aeruginosa* isolate collections

The SickKids cohort consisted of clinical *P. aeruginosa* isolates (*N* = 92) from sputum samples obtained from 67 pediatric CF patients undergoing eradication treatment with inhaled tobramycin for new-onset *P. aeruginosa* infection at SickKids (Toronto, Canada) from 2011 to 2018 (ongoing)^11,46^. New-onset infection was defined as a sputum culture positive for *P. aeruginosa* with at least 3 *P. aeruginosa* negative respiratory tract cultures in the prior 12 months. Eradication was defined based on a *P. aeruginosa* negative culture of the first respiratory tract specimen taken after the end of inhaled tobramycin therapy (1 week post end of treatment). The initial *P. aeruginosa* isolates were cultured, before antibiotic treatment, from patients in whom *P. aeruginosa* was successfully eradicated with inhaled tobramycin (eradicated isolates) and from patients in whom eradication therapy failed (persistent isolates).

In addition, we also used the larger *P. aeruginosa* isolate collection (*N* = 194) from the EPIC trial of early eradication (2004–2009)^25^. In this randomized trial, four anti-pseudomonal treatment regimens were compared in 304 children with CF with new-onset *P. aeruginosa* infection (at least 2 years of *P. aeruginosa* negative respiratory tract cultures). The subset of subjects who most closely resembled the SickKids population were the 37 children with CF who were *P. aeruginosa* positive at baseline (week 0), had not received prior antibiotic treatment and subsequently received 28 days of inhaled tobramycin (within 1 month of the initial screen). In contrast to the SickKids cohort, in the EPIC cohort, *P. aeruginosa* isolates were cultured from oropharyngeal swabs rather than sputum and eradication was defined as a *P. aeruginosa* negative culture 10 weeks following the end of treatment. Experiments were repeated in this secondary *P. aeruginosa* collection, comparing baseline *P. aeruginosa* isolates in a blinded fashion, to determine the generalizability of results.

### Psl dot blot analysis

*P. aeruginosa* isolates, PAO1 and *Δpsl* (Psl deletion strain (PAO1*ΔpslBCD*) were grown overnight in lysogeny broth (LB) with shaking (225 rpm). The overnight culture was diluted 1:100 and grown for 3 h at 37 °C to obtain an OD of 0.1 at 600 nm (early log phase). Of this culture, 1 mL was transferred to a Sarstedt 96-well Tissue Culture plate (Thermo Fisher Scientific, Mississauga, ON) and grown for 48 h at 37 °C with LB replacement after the first 24 h. The wells were washed 1× with phosphate-buffered saline (PBS) and 200 µL of PBS was added back into the wells. The cell-attached fraction of the wells was disrupted and the optical density (OD) of the cell suspension in PBS was normalized to 0.2 at 600 nm, followed by centrifugation (3000 × *g*, 4 ^o^C, 15 min).

The centrifuged product consisted of a cell pellet and supernatant. The cell pellet was re-suspended in 500 µL of 0.9 % NaCl and lysed by bath sonication (VWR Ultrasonic 50D, 5 × 2 min)^16^ with 2 min cooling on ice after each interval. The supernatant (pre-lysis) and lysate (post-lysis) were loaded into the Minifold II Slot-Blot Manifold system (50 µL each) and spotted on a nitrocellulose membrane. The blots were air dried and blocked with 2% bovine serum albumin (BSA), followed by 24 h incubation at 4 °C. The blots were rinsed 3× with 1× tris buffered saline with Tween 20 (TBST) and incubated with 1:5000 primary anti-Psl antibody in 1× TBST for 1 h at room temperature (RT). The primary antibody was decanted and the blots were rinsed 3× with 1× TBST, then incubated with 1:3000 secondary goat anti-rabbit IgG antibody (Bio-Rad, Mississauga, ON) for 1 h at RT. The secondary antibody was decanted and the blots were rinsed 3× with 1× TBST, after which 50 µL of 1× TBST was added back onto the blots to prevent drying. Bound antibody was detected using a SuperSignal West Atto chemiluminescent substrate (Thermo Fisher Scientific, Mississauga, ON). Blots were imaged using the ChemiDoc Imaging System and variations in signal intensities were quantified with signal accumulation mode. The mean intensity value for each isolate (done in triplicate) was calculated as arbitrary units (a.u).

### Confocal imaging of *P. aeruginosa* biofilms

*P. aeruginosa* isolates were grown in Nunc Lab-Tek II, 8-chambered cover glass slides (Thermo Fisher Scientific, Mississauga, ON) as previously described^13^. *P. aeruginosa* was grown in LB overnight at 37 °C on a shaker (225 rpm). The overnight culture was diluted 1:100 and grown to an OD of 0.1 at 600 nm (early log phase), after which 220 µL of the culture was seeded into an 8-chambered cover glass slide (Thermo Fisher Scientific, Mississauga, ON) and incubated at 37 °C.

To measure antibody binding alone, after 48 h of growth, media was removed and fluorescently labeled anti-Psl antibody (56 µg/mL) was added for 90 min. Media was then removed from the wells and 200 µL of Syto-9^TM^ live-cell fluorescent stain (Thermo Fisher Scientific) was added and incubated for 45 min at RT. The wells were then gently washed 2× with LB and 200 µL of LB was added back into the wells prior to confocal laser scanning microscopy (CLSM). This procedure was repeated in triplicates for each *P. aeruginosa* isolate.

To measure antibody binding in the presence of tobramycin, after 24 h of growth, media was removed and replaced with fluorescently labeled anti-Psl antibody (56 µg/mL) for 1 h at RT. Then, either 100 µL of LB alone or LB with tobramycin (final concentration 1000 µg/mL based on achievable aerosolized concentrations in CF patients^38^) was added to designated wells and incubated for a total of 24 h at 37 °C. Media was then removed from the wells and 200 µL of Syto-9^TM^ live-cell fluorescent stain (Thermo Fisher Scientific) was added and incubated for 45 min at RT. The wells were then gently washed 2× with LB and 200 µL of LB was added back into the wells prior to CLSM. This procedure was repeated in triplicates for each *P. aeruginosa* isolate.

Images were acquired using a Quorum Wave FX Borealis Spinning Disk Confocal Microscope, based on a Yokohama CSU-10 scan head. Fluorescently labeled (red) anti-Psl antibody and live cell (green) stains were excited using 561 and 491 nm excitation lines, respectively, and visualized with a 25×/0.80 Zeiss water immersion lens, coupled to a 1.6× magnification coupler. Image acquisition was performed using the Quorum Volocity 6.3 software. A total of 6 z-stack images were acquired per well in increments of 0.3 µm. Anti-Psl binding within *P. aeruginosa* biofilms was calculated using Volocity software to quantify total volume of voxels in the green and red channels. Quantification of *P. aeruginosa* biovolume intensity was performed using Volocity software. Surface-to-biovolume ratio, as a measure of *P. aeruginosa* aggregation, was quantified as previously described, using Comstat2 software as a plugin to ImageJ^48^. All images are included in the Supplemental materials as Supplemental Fig. 4.

### Bacterial counts of *P. aeruginosa* biofilms

Colony-forming units (CFU) counts were determined by growing *P. aeruginosa* biofilms in cation-adjusted Mueller Hinton broth (CAMHB) in 8-chambered cover glass slides (Thermo Fisher Scientific, Mississauga, ON), under the same conditions previously described herein for confocal imaging. After 24 h growth with subsequent 24 h growth with exposure to antibodies and tobramycin, media was removed, washed 2× with PBS, and 500 µL of fresh PBS was added into the wells. The cell-attached fraction of the wells were disrupted and cell suspensions were serial diluted, then plated on blood agar. Plates were grown for 24 h at 37 °C and CFUs were counted. This procedure was repeated in triplicates for each *P. aeruginosa* isolate, reporting mean CFU counts (log transformed) as previously described^49^.

### ATP assays of *P. aeruginosa* biofilms

Cell metabolic activity was assessed using a modified ATP assay method for biofilms^50,51^. In brief, *P. aeruginosa* was grown overnight in CAMHB (Sigma-Aldrich, Oakville, ON) at 37 °C with shaking (225 rpm). The overnight culture was diluted 1:100 and grown to an OD of 0.1 at 600 nm. Of this log phase culture, 220 µL was added to 8 wells of a white Greiner Medium Binding 96-well plate (Sigma-Aldrich, Oakville, ON) and incubated for 24 h at 37 °C. After 24 h, media was removed and the wells were washed 2× with CAMHB. Subsequently, 100 µL of anti-Psl antibody (56 µg/mL) was added to designated wells and incubated for 1 h at RT. Either 100 µL of CAMHB alone or CAMHB with tobramycin (final concentration 1000 µg/mL) was added to designated wells and incubated for 24 h at 37 °C. Media was then removed and the wells were washed 3× with CAMHB. Finally, to measure ATP in the cell-attached fraction of the wells, 100 µL of Bac Titer-Glo^TM^ reagent (Promega, Madison, WI) was added. The cell-attached fraction of the wells was disrupted and the plate was gently mixed on an orbital shaker for 10 min prior to luminescence reading as per the manufacturer’s protocol. This procedure was repeated in triplicates for each *P. aeruginosa* isolate.

### Psl gene polymorphisms

To determine whether there were mutational differences in the Psl genes of persistent versus eradicated isolates, we analyzed the gene region from 2453667-2472076 spanned by the 14 *psl* genes, *pslA, pslB, pslC, pslD, pslE, pslF, pslG, pslH, pslI, pslJ, pslK, pslL, pslM, pslN* for polymorphisms in each of the seven persistent and seven eradicated SickKids *P. aeruginosa* strains. Whole genome sequence data for each of the strains was previously collected via DNA extraction and sequencing^13^. The data for each strain was then mapped to PAO1 as a reference, and single nucleotide polymorphisms called via Snippy^52^. We identified non-synonymous (missense) mutations found in each *psl* locus and compared polymorphism levels between persistent and eradicated strains on a per gene (and across all genes) basis and for the entire Psl region. All data are available through Bioproject PRJNA556419 with the following Genbank accessions: JAGHMW000000000 (Pa50), JAGHMV000000000 (Pa263), JAGHMU000000000 (Pa288), JAGHMT000000000 (Pa325), JAGHMS000000000 (Pa342), JAGHMR000000000 (Pa375), JAGHMQ000000000 (Pa380), JAGHMP000000000 (Pa404), JAGHMO000000000 (Pa505), JAGHMN000000000 (Pa551), JAGHMM000000000 (Pa558), JAGHML000000000 (Pa565), JAGHMK000000000 (Pa580), JAGHMI000000000 (Pa549).

### Anti-CdrA western blot analysis of static biofilms

Sample preparation for western blot analysis was performed as previously described^27^. Briefly, static biofilms were cultured in 6-well plates using TSB for 24 h at 30 °C. Biofilms were then harvested by passage through an 18-gauge syringe. Biofilms were normalized by OD 600 nm. Biofilms were separated by centrifugation into supernatant and cellular fractions. Samples were then analyzed by western blot analysis as previously described^36^ using an anti-CdrA antibody raised against CADGKTKVYGDADPS (Genscript). This amino acid sequence is a conserved stretch of amino acids in the C-terminal region of the protein.

### Statistical analysis and institutional approvals

All statistical analyses were done using GraphPad 5.0. Continuous variables were compared using non-parametric Mann–Whitney test; *p*-value of <0.05 was considered significant. This study was approved by the SickKids Research Ethics Board (REB#1000065841) with waiver of consent due to de-identified nature of bacterial isolates.

### Reporting summary

Further information on research design is available in the Nature Research Reporting Summary linked to this article.

## Supplementary information

Supplemental Materials

Reporting Summary

## Data Availability

The data that support the findings of this study are available from the corresponding author upon reasonable request. All data are available through Bioproject PRJNA556419 for the above mentioned Genbank accessions.
