# Supplementary material for: The role of Psl in the failure to eradicate Pseudomonas aeruginosa biofilms in children with cystic fibrosis
Source: NPJ Biofilms Microbiomes. 2021 Aug 4;7:63. doi: 10.1038/s41522-021-00234-3 (PMC8338932; doi:10.1038/s41522-021-00234-3)

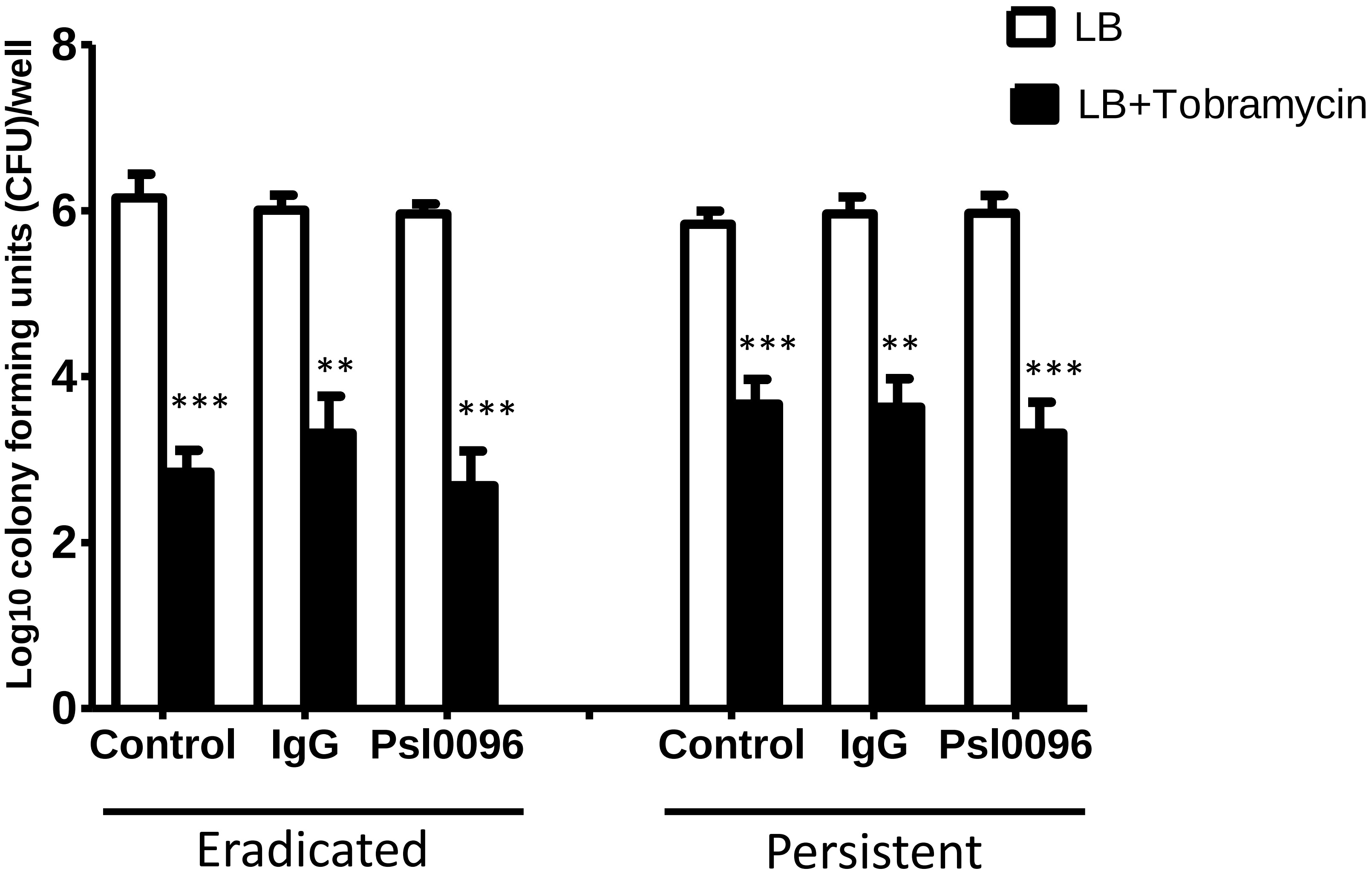

Supplemental Figure 1. SickKids *P. aeruginosa* isolates (N=7 Persistent; N=7 Eradicated) were grown as biofilms for 24 hrs after which antibodies (LB control, IgG or anti-Psl 0096) and tobramycin (or LB alone) were added for the following 24 hrs before plating for colony counts. The mean colony forming unit (CFU) per well for each isolate (done in triplicate) was calculated and the log transformed (log_{10}) mean of all isolates was plotted with standard error of the mean (SEM). Comparisons with and without tobramycin treatment were performed for each condition using non-parametric Mann-Whitney test ***$p<0.001$, **$p<0.01$

Strain	E/P	pslA	pslB	pslC	pslD	pslE	pslF	pslG	pslH	pslI	pslJ	pslK	pslL	pslM	pslN	pslO
**263**	E	0	1	1	0	0	2	0	1	1	0	3	2	1	1	0
**288**	E	0	1	1	0	0	2	0	1	1	1	1	1	2	1	0
**325**	E	0	2	2	0	1	1	0	3	2	0	2	0	1	0	1
**549**	E	0	0	1	0	0	2	0	1	1	1	1	1	1	1	0
**558**	E	0	0	1	0	0	2	0	1	1	1	1	1	1	1	0
**404**	E	0	2	2	0	1	1	0	3	2	0	2	0	1	0	1
**50**	E	0	0	1	0	0	0	0	0	0	0	0	0	1	0	0
**375**	P	0	1	3	0	1	1	1	2	3	0	1	2	2	0	1
**551**	P	0	0	1	0	0	2	0	1	1	1	1	1	1	1	0
**580**	P	0	1	1	0	0	2	0	1	1	0	3	2	1	1	0
**380**	P	0	1	3	0	1	1	0	1	1	0	1	1	2	0	3
**342**	P	0	0	1	0	0	2	0	1	1	1	1	1	1	1	0
**505**	P	0	1	2	0	0	2	0	2	1	0	0	1	2	0	1
**565**	P	1	0	2	0	1	1	1	1	1	0	1	0	2	0	1
	**Ttest**	0.337	0.484	0.165	N/A	0.611	0.698	0.147	0.765	0.718	0.611	0.580	0.290	0.109	0.626	0.223

Supplemental Figure 2. The number of non-synonymous mutations in each Psl gene was counted for each of the 14 strains. T-test was used to determine whether there was a significant difference in the number of mutations between all 14 of the strains for each Psl gene as well as when all the Psl genes were considered together.

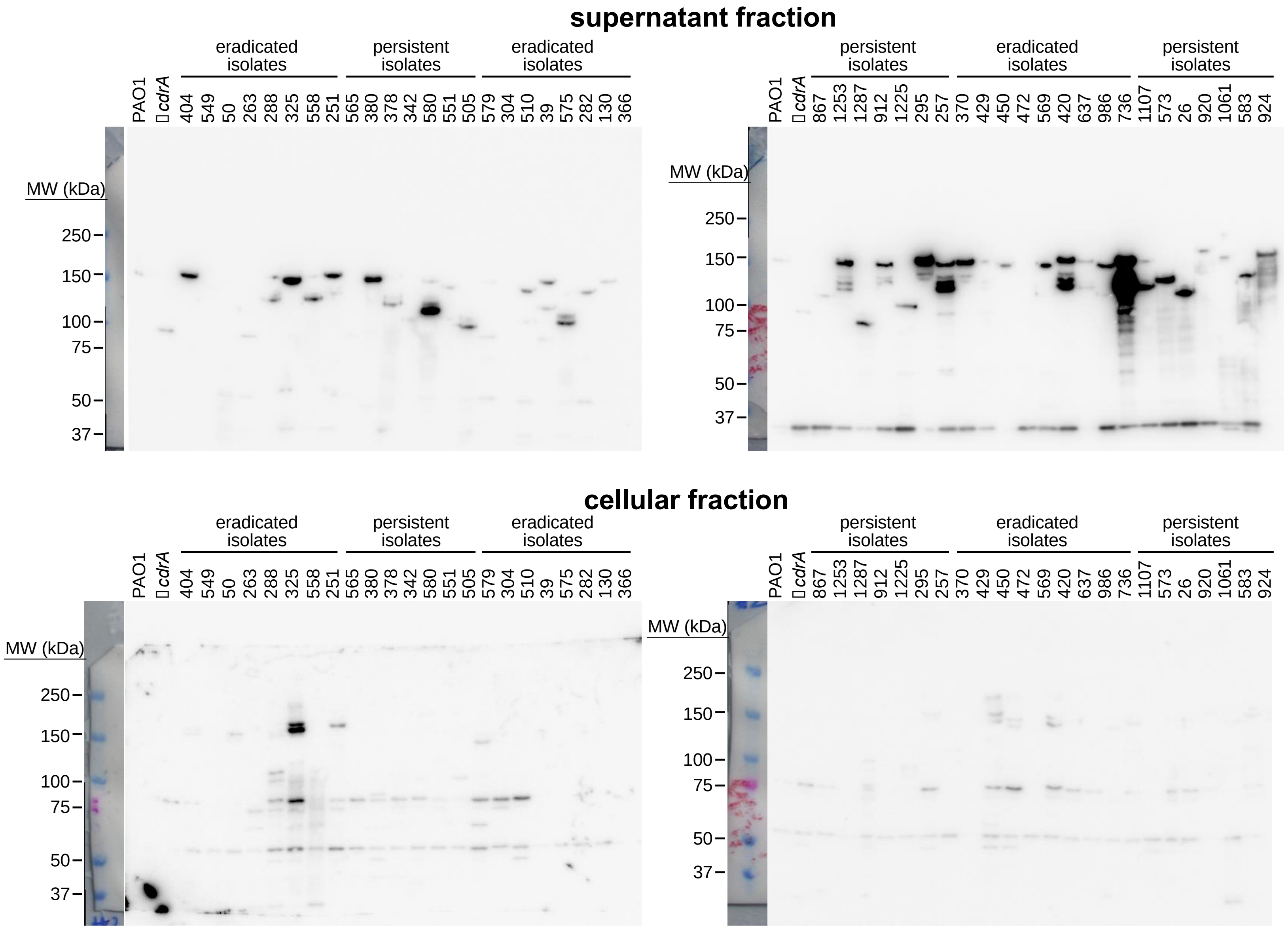

Supplemental Figure 3. SickKids *P. aeruginosa* isolates (N=21 Persistent; N=25 Eradicated) were grown as biofilms for 24 h then separated by centrifugation into supernatant and cellular fractions. Samples were then analyzed by western blot analysis using an anti-CdrA antibody. PAO1 was used as a positive control and the CdrA deletion strain (PAO1*ΔcdrA*) was used as a negative control. All blots derive from the same experiment and were processed in parallel.

Supplementary Figure 4

Pa342 (Persistent) Trial1

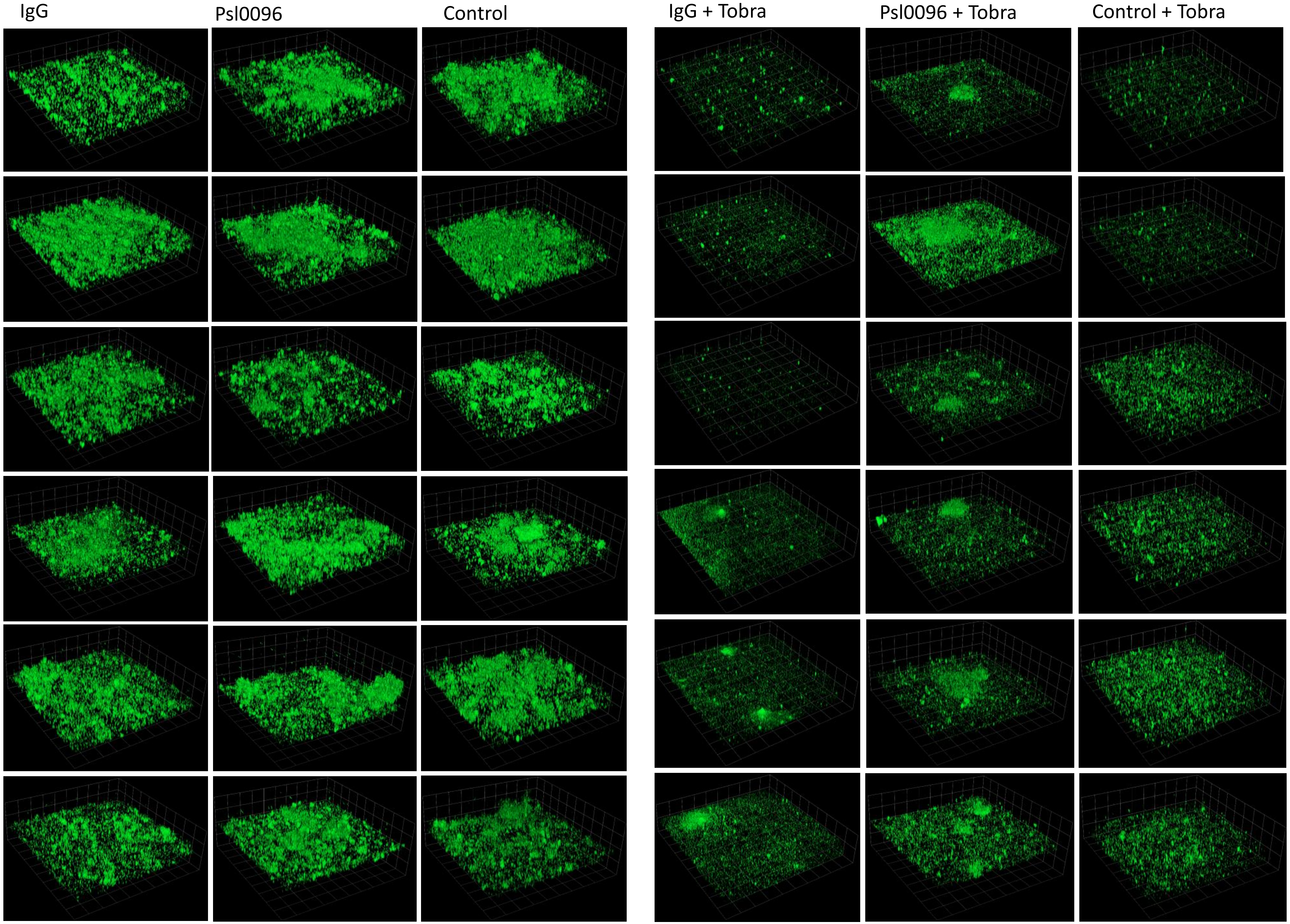

Pa342 (Persistent) Trial2

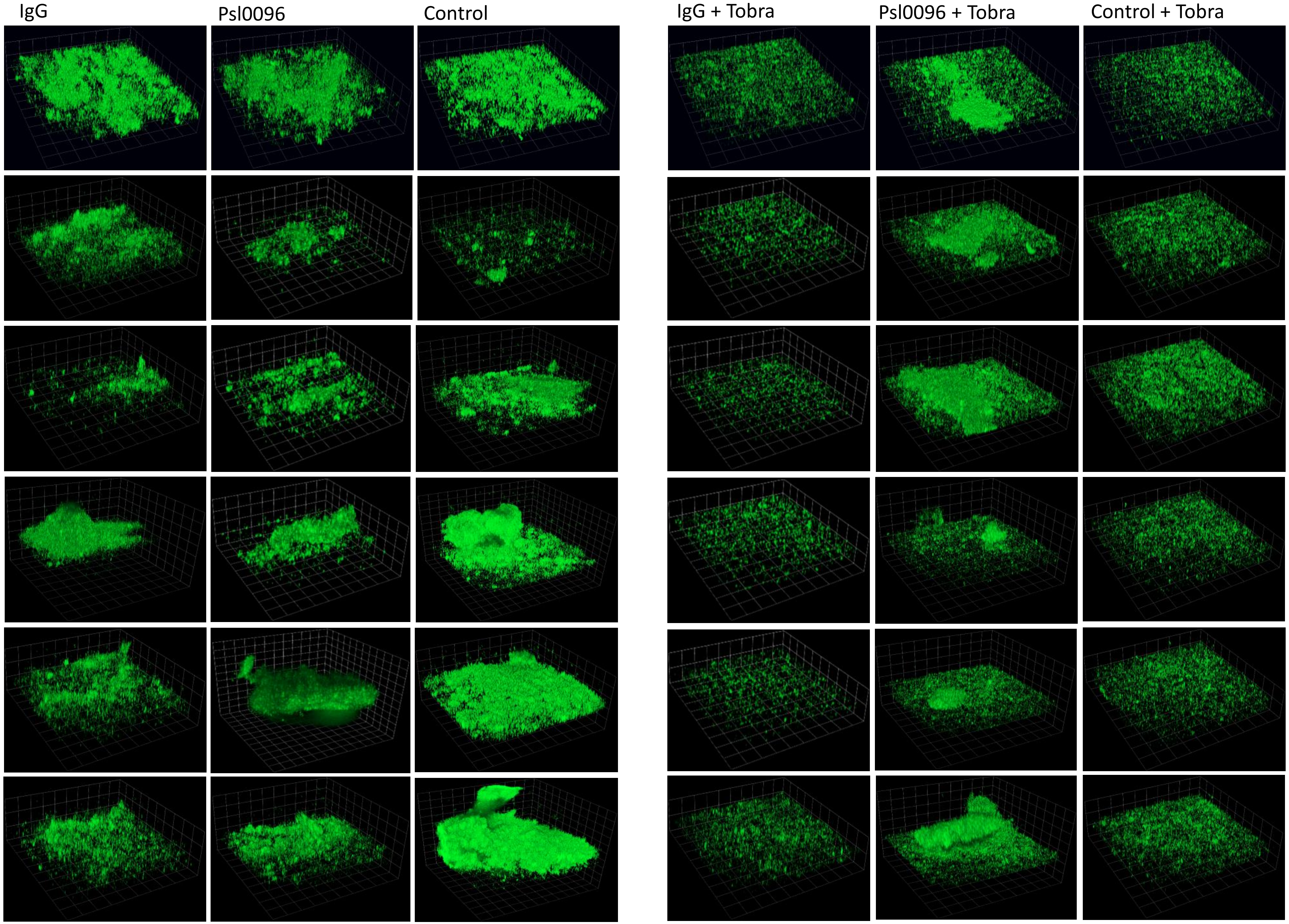

Pa342 (Persistent) Trial3

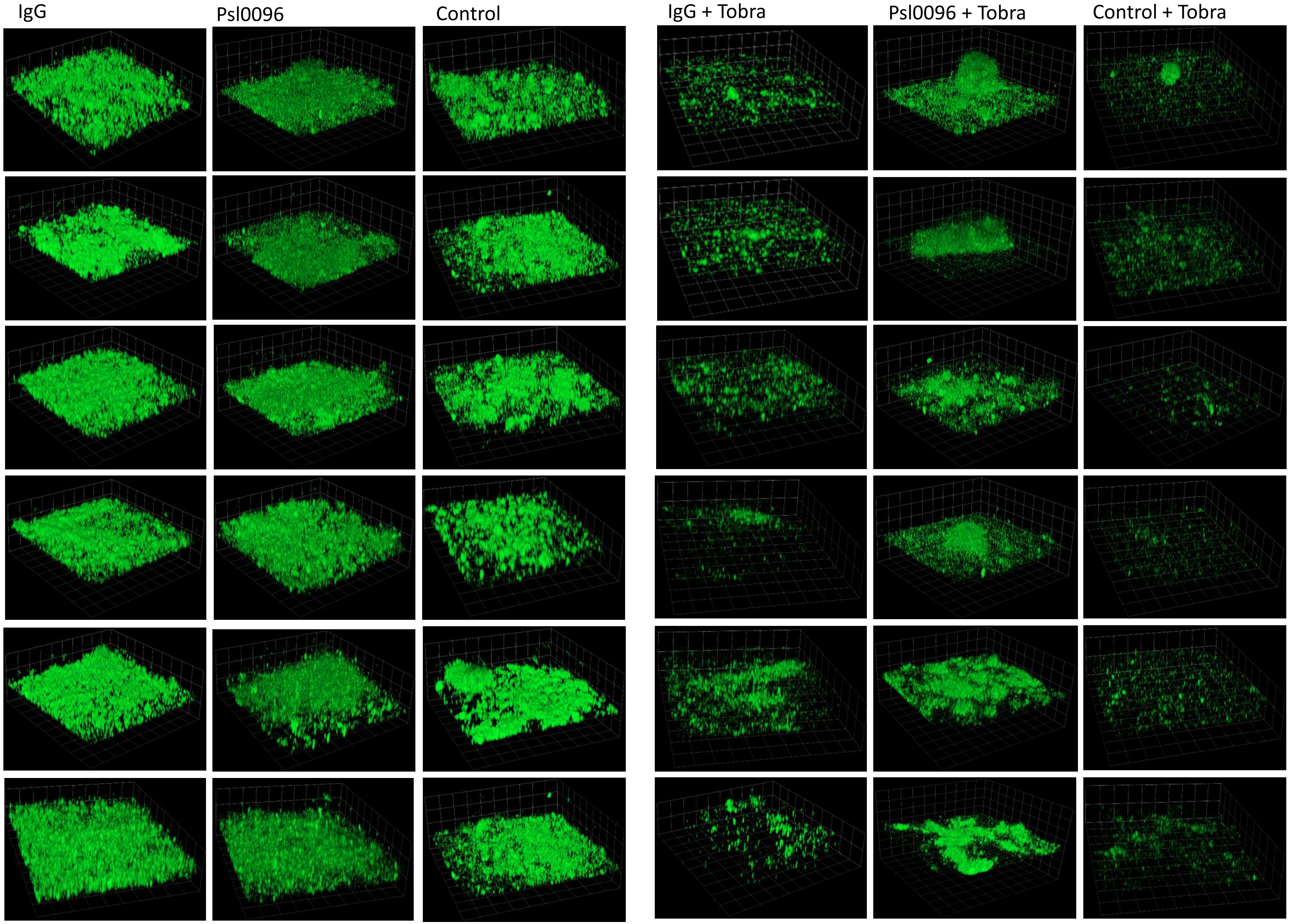

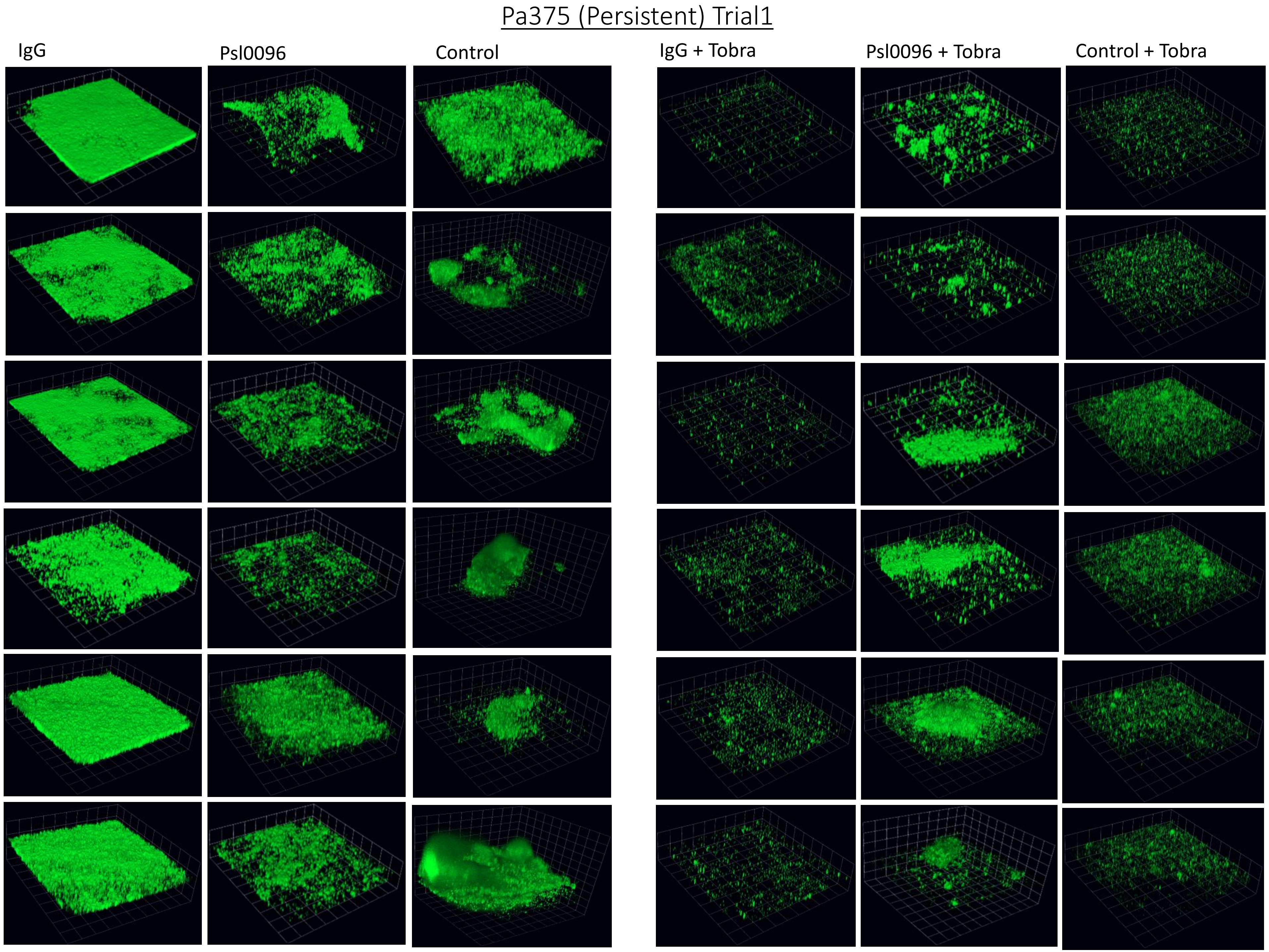
Pa375 (Persistent) Trial1
IgG
Psl0096
Control
IgG + Tobra
Psl0096 + Tobra
Control + Tobra

Pa375 (Persistent) Trial2

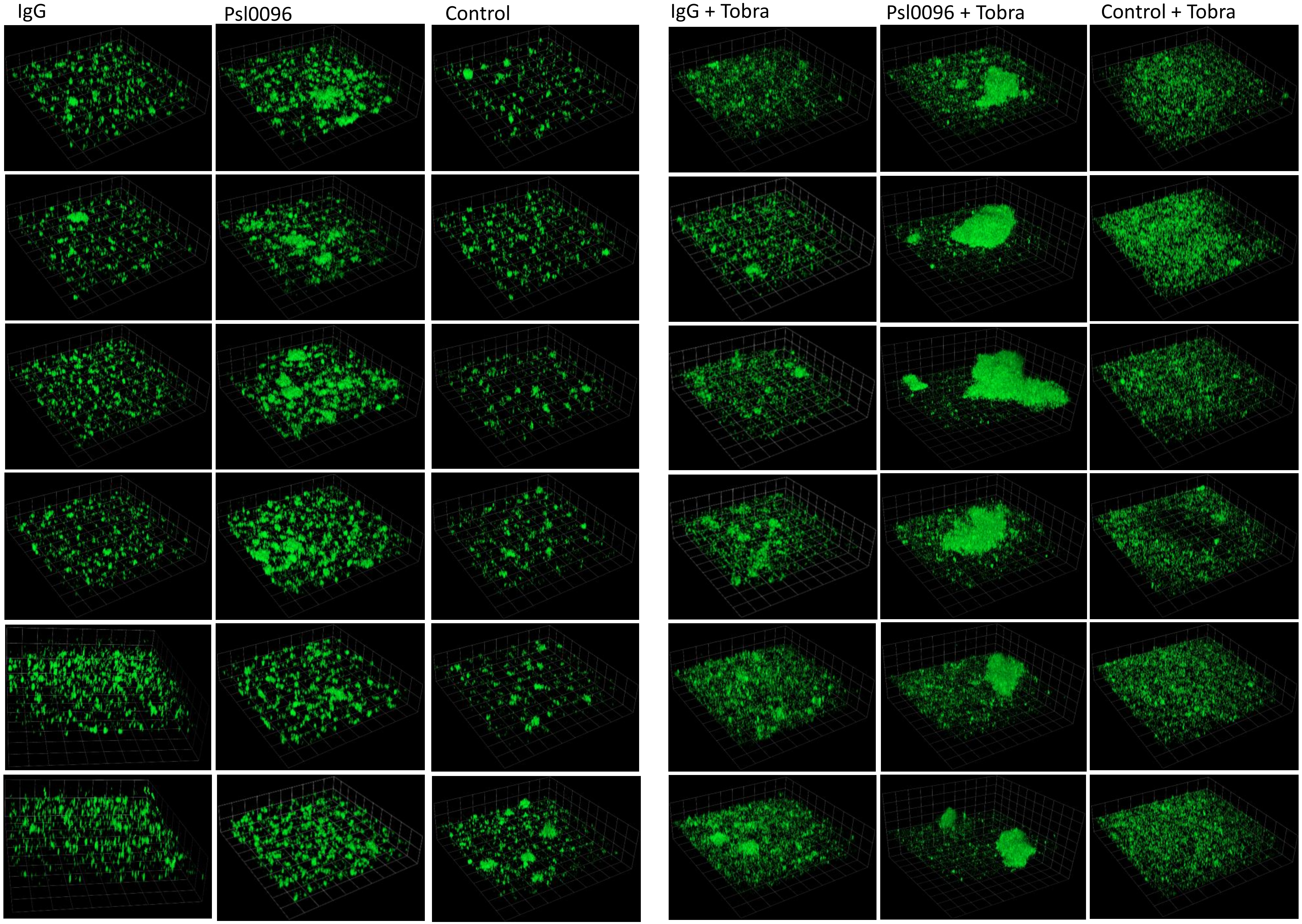

Pa375 (Persistent) Trial3

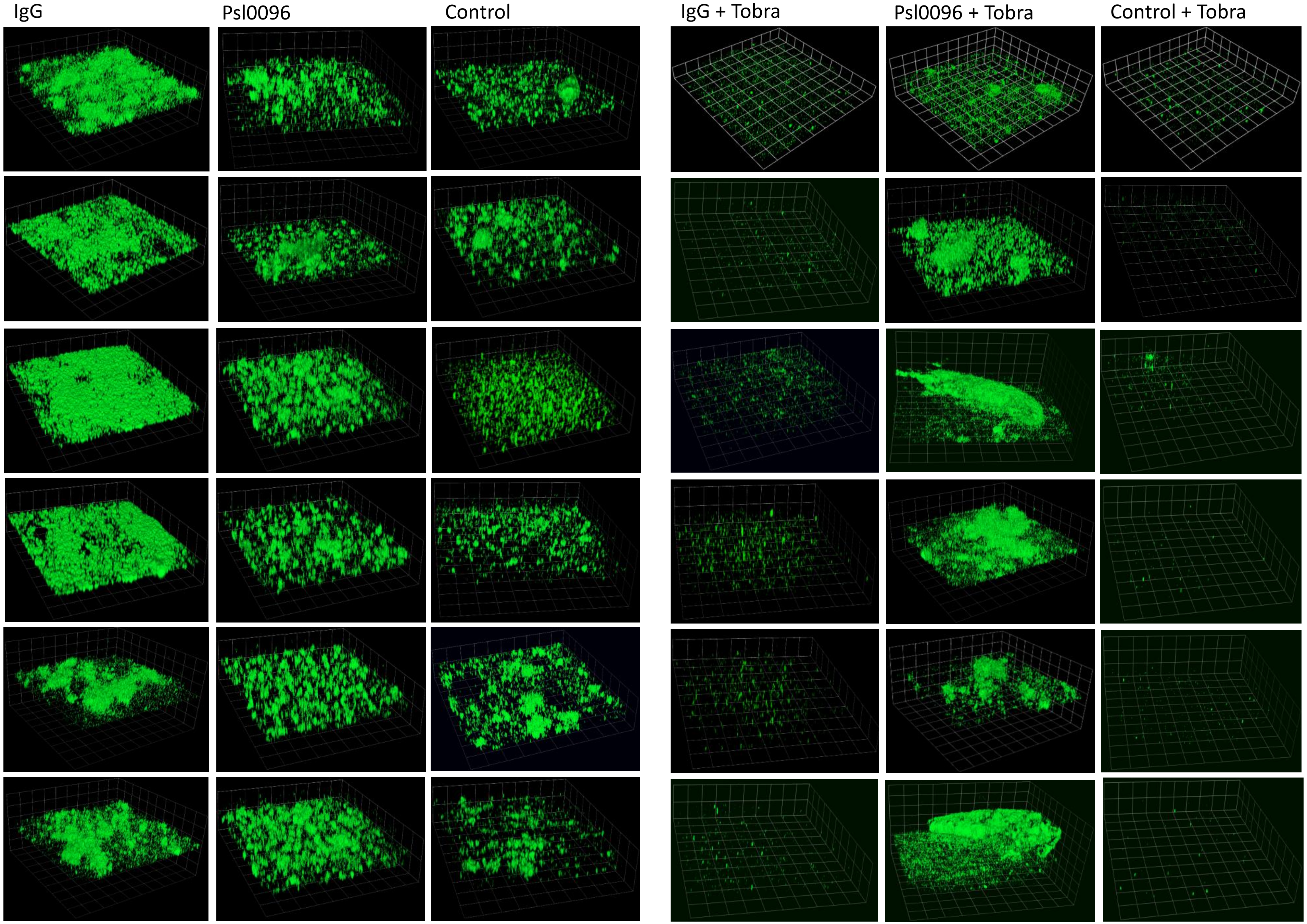

Pa380 (Persistent) Trial1

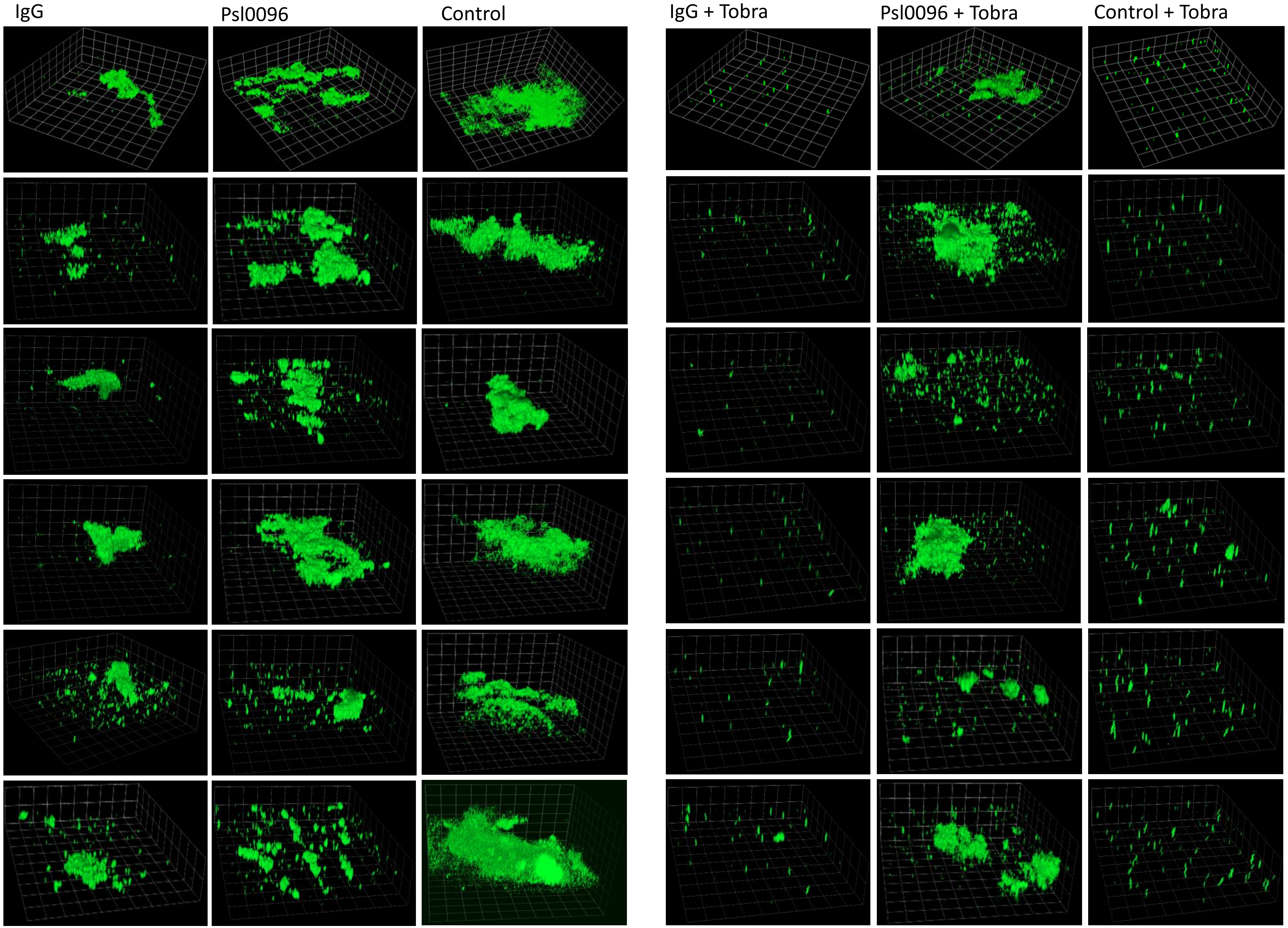

Pa380 (Persistent) Trial2

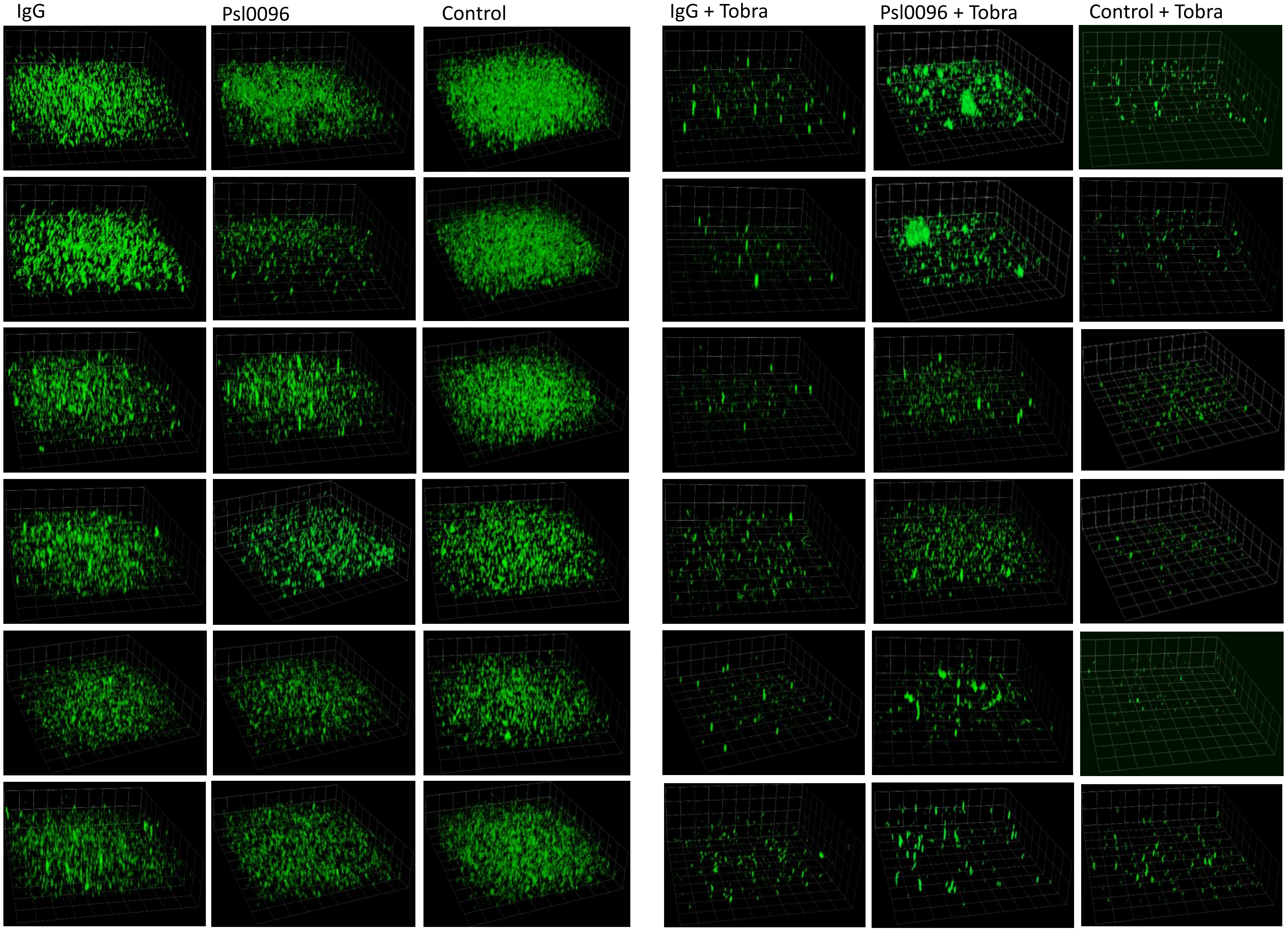

Pa380 (Persistent) Trial3

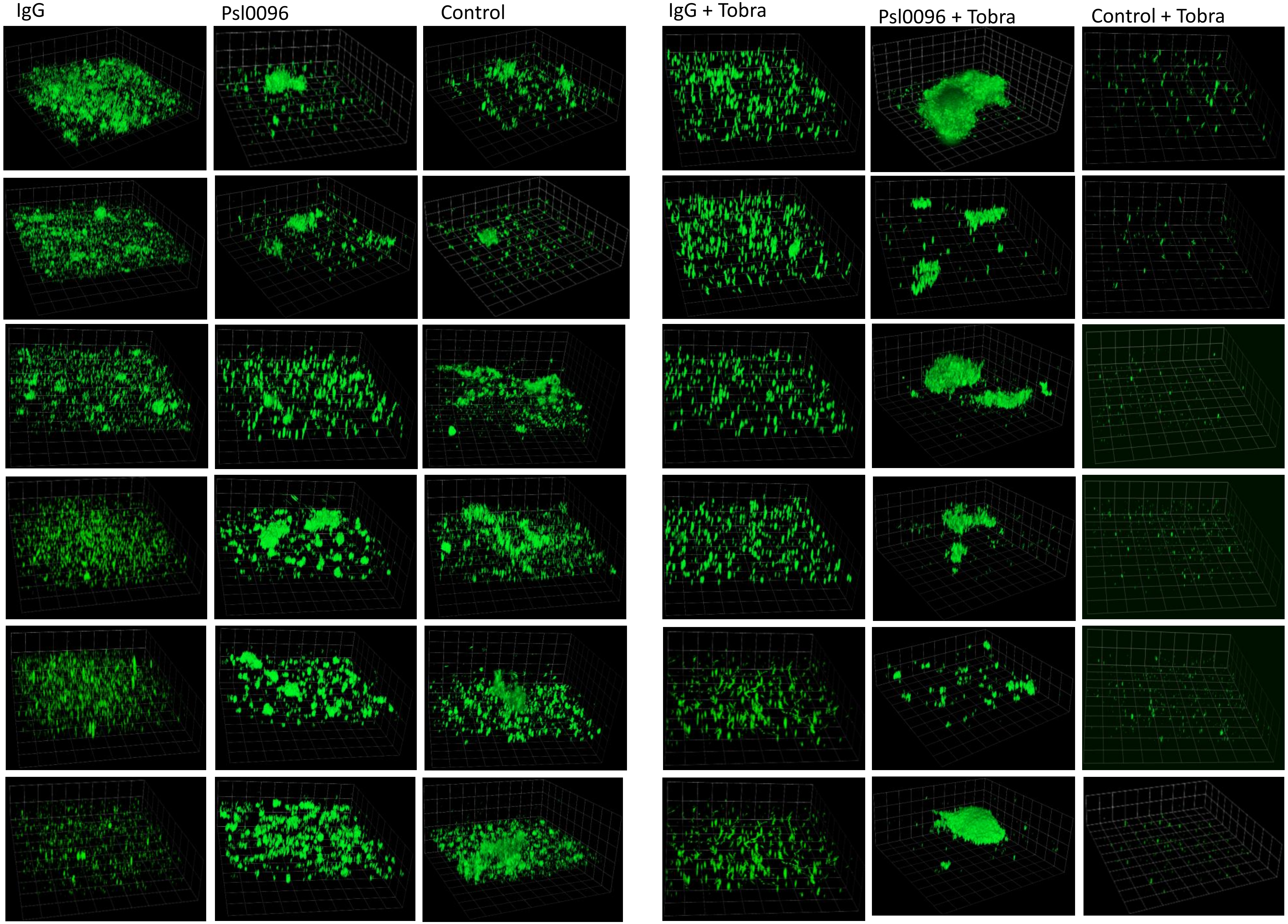

Pa565 (Persistent) Trial1

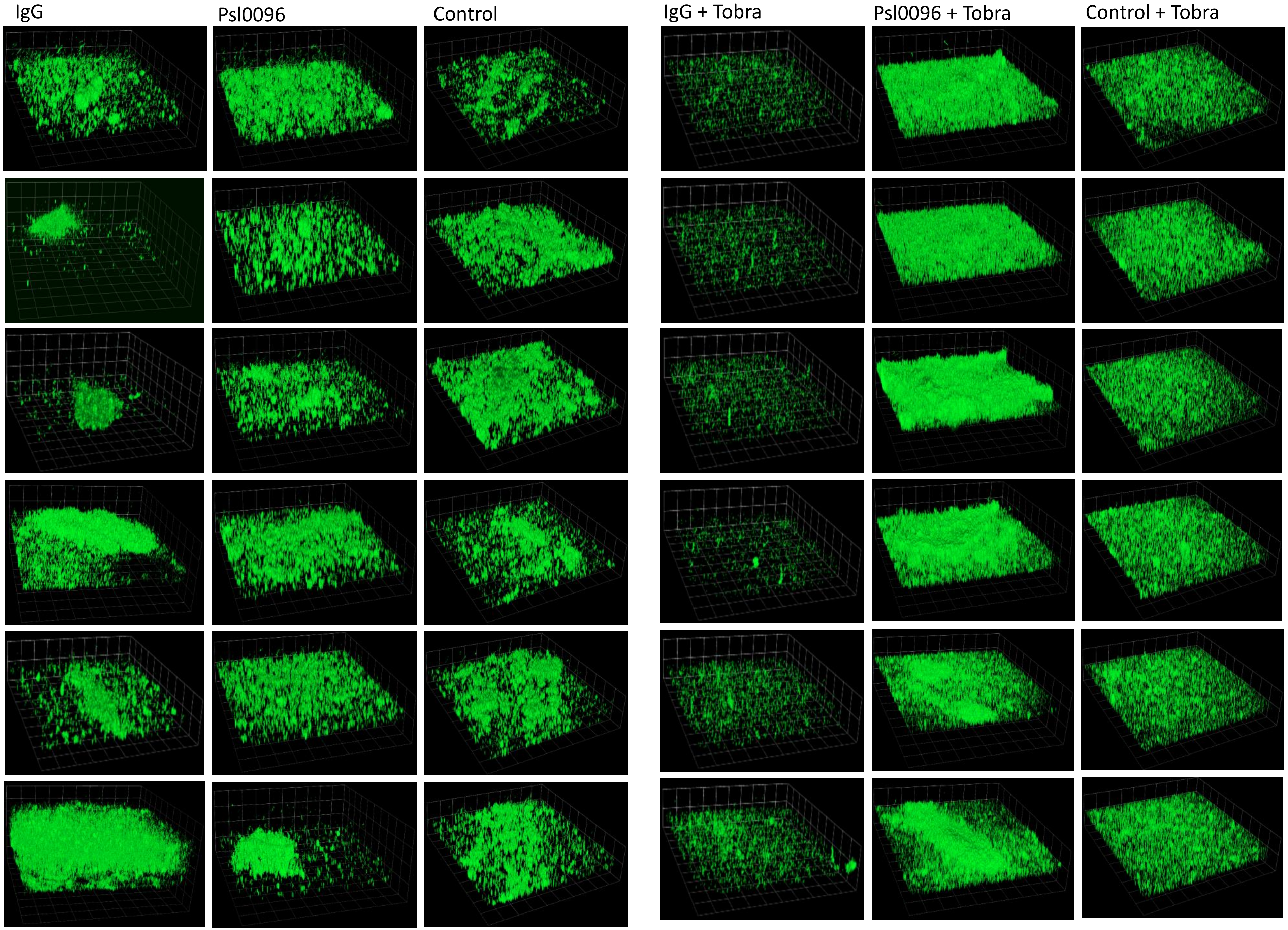

Pa565 (Persistent) Trial2

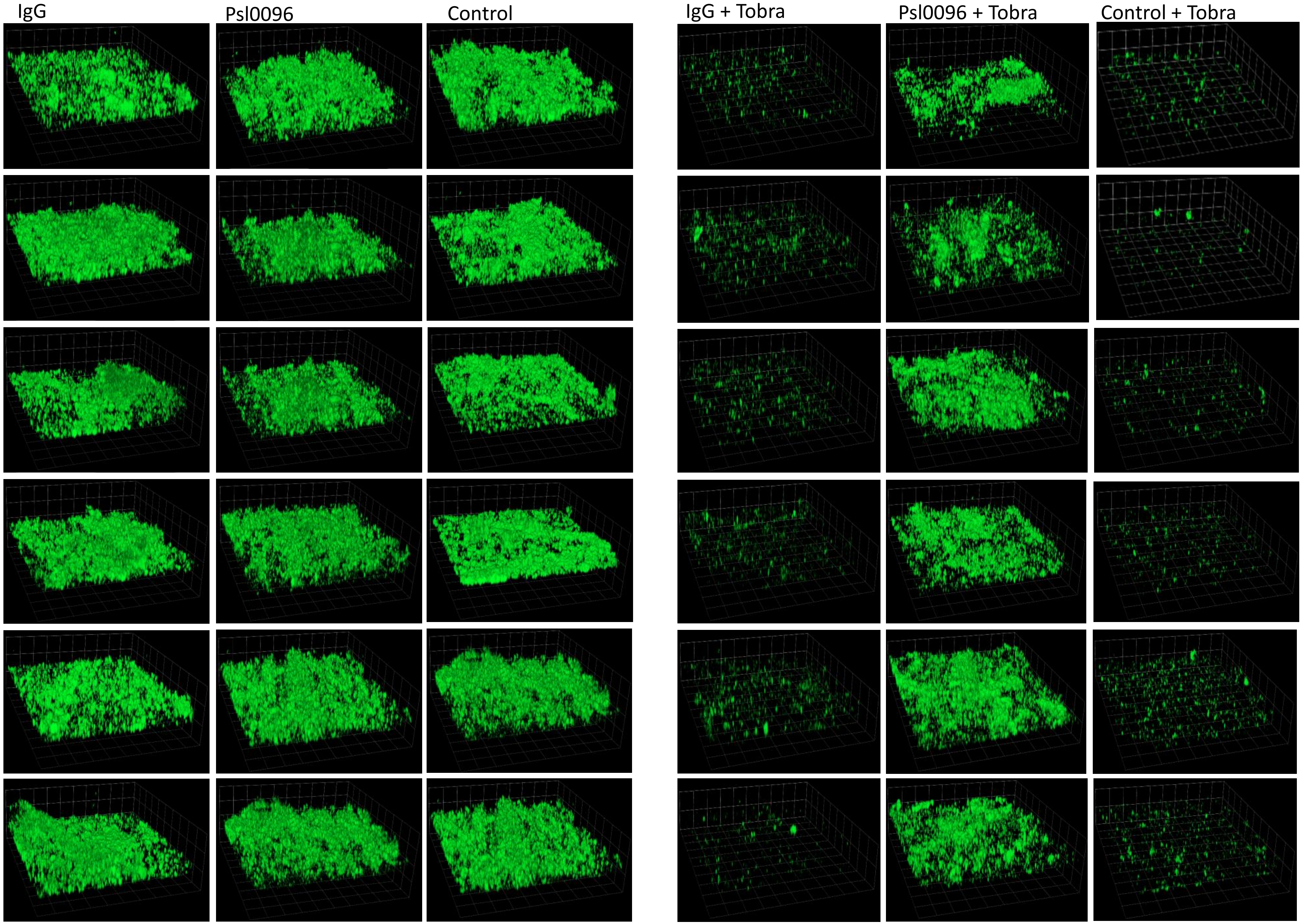

Pa565 (Persistent) Trial3

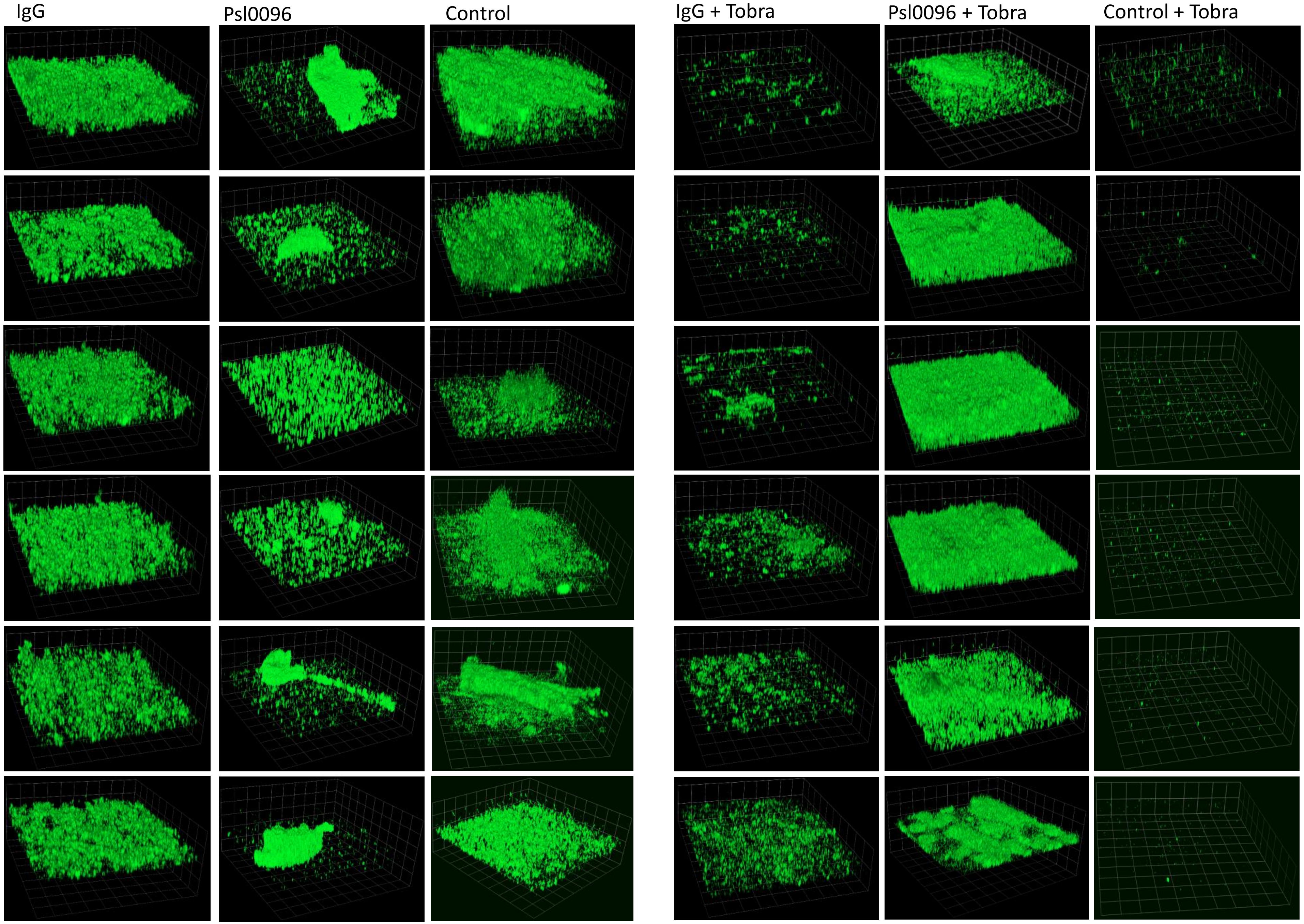

Pa580 (Persistent) Trial1

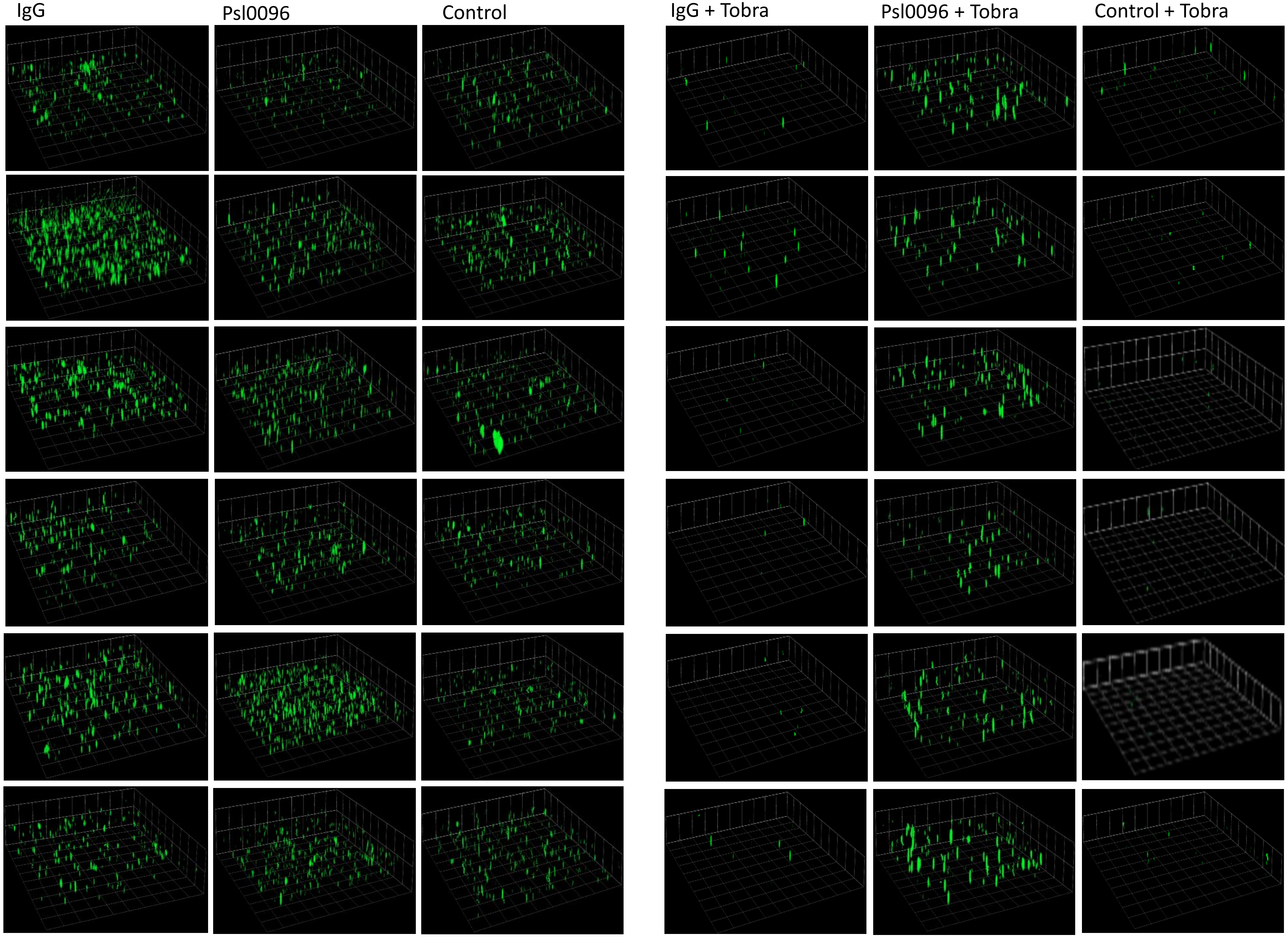

Pa580 (Persistent) Trial2

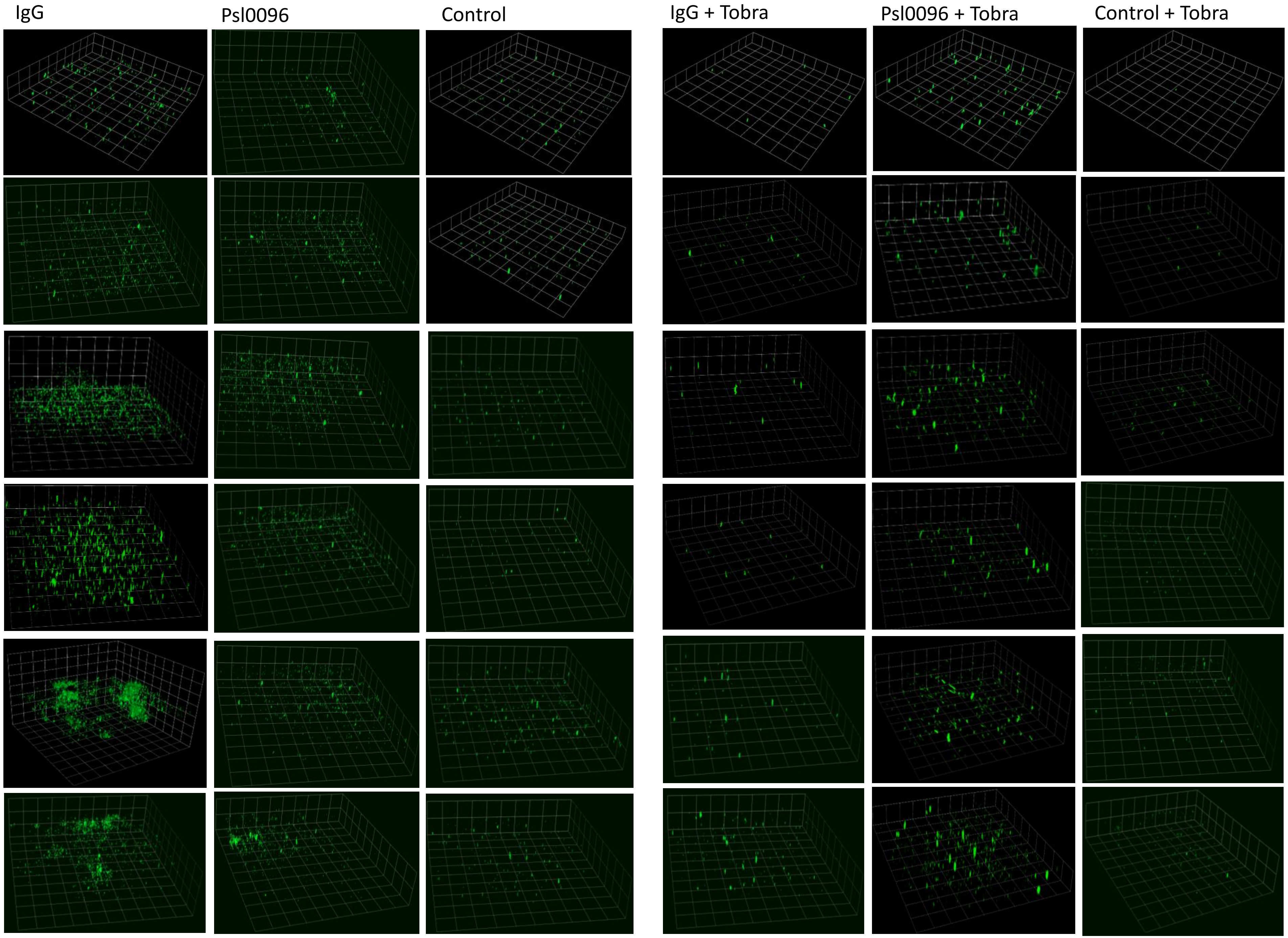

Pa580 (Persistent) Trial3

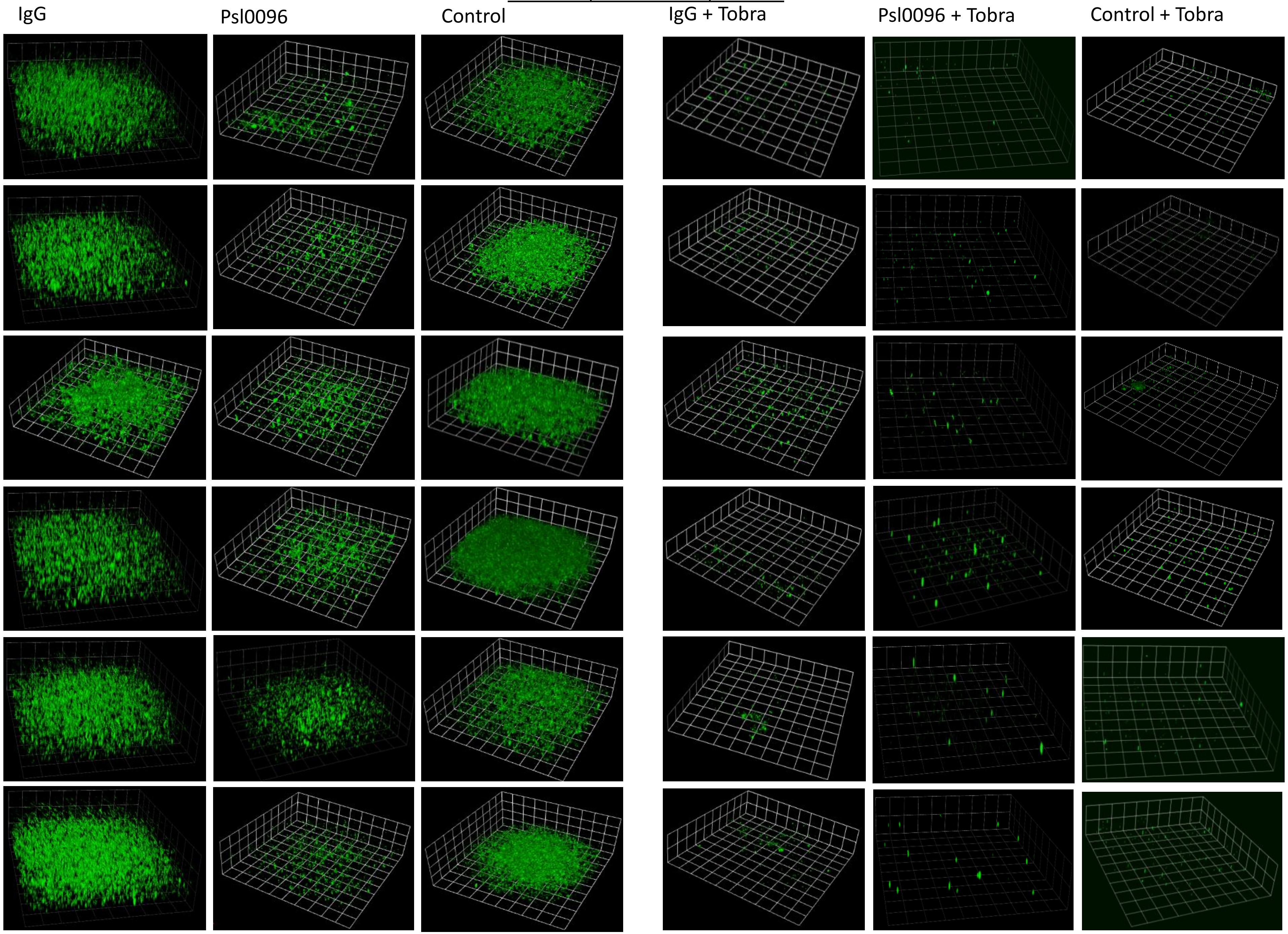

Pa505 (Persistent) Trial1

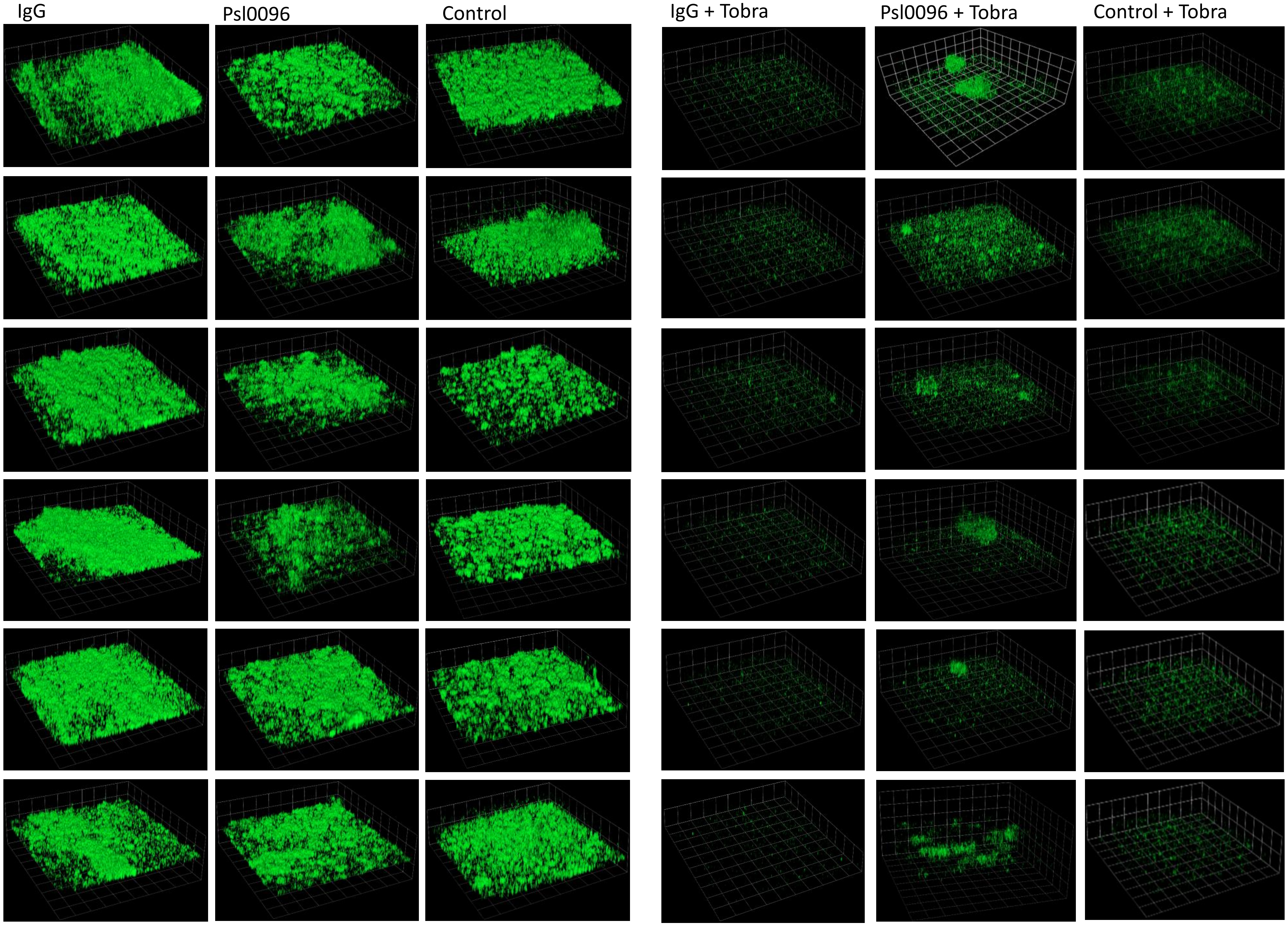

Pa505 (Persistent) Trial2

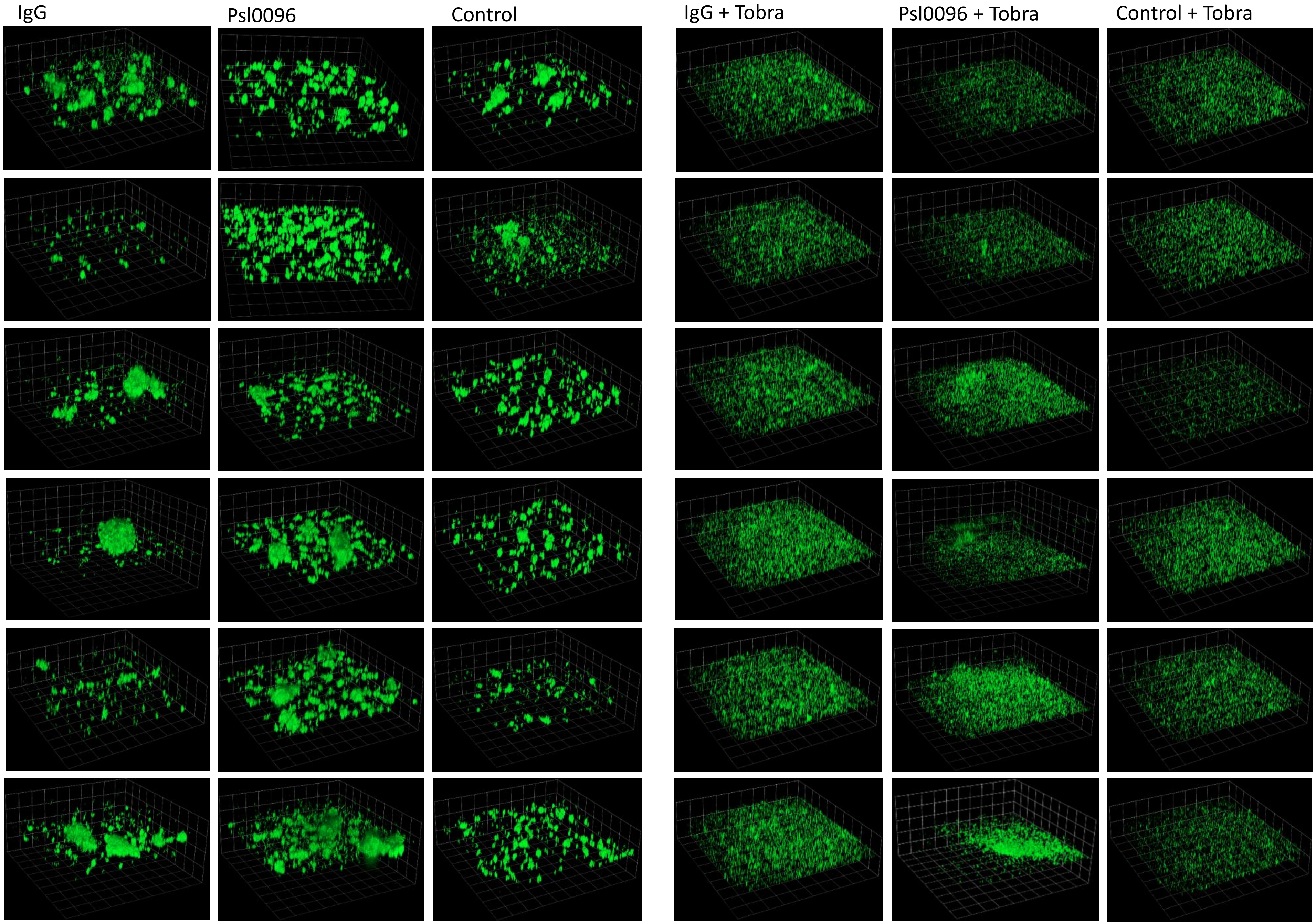

Pa505 (Persistent) Trial3

IgG | Psl0096 | Control | IgG + Tobra | Psl0096 + Tobra | Control + Tobra

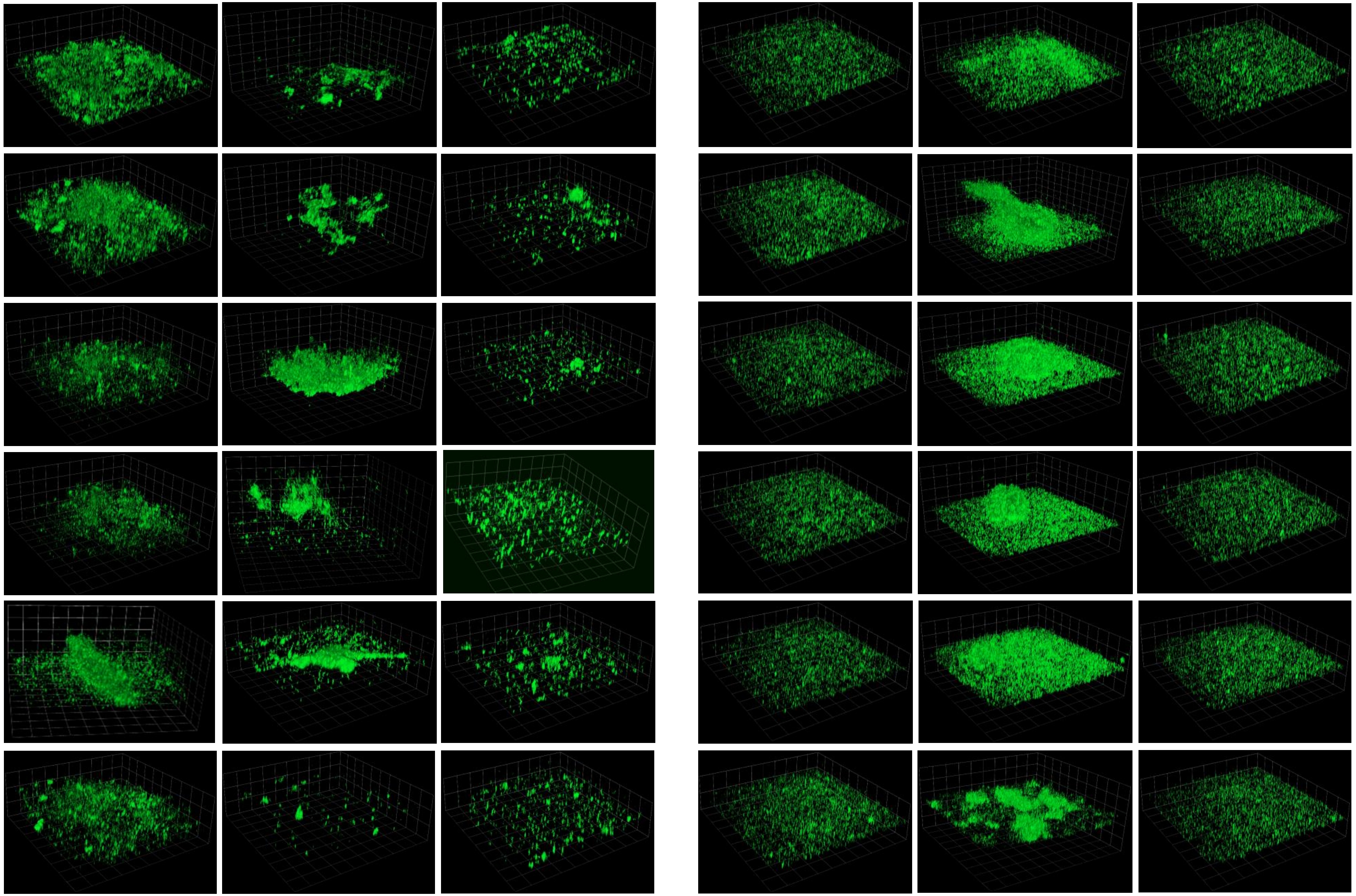

Pa551 (Persistent) Trial1

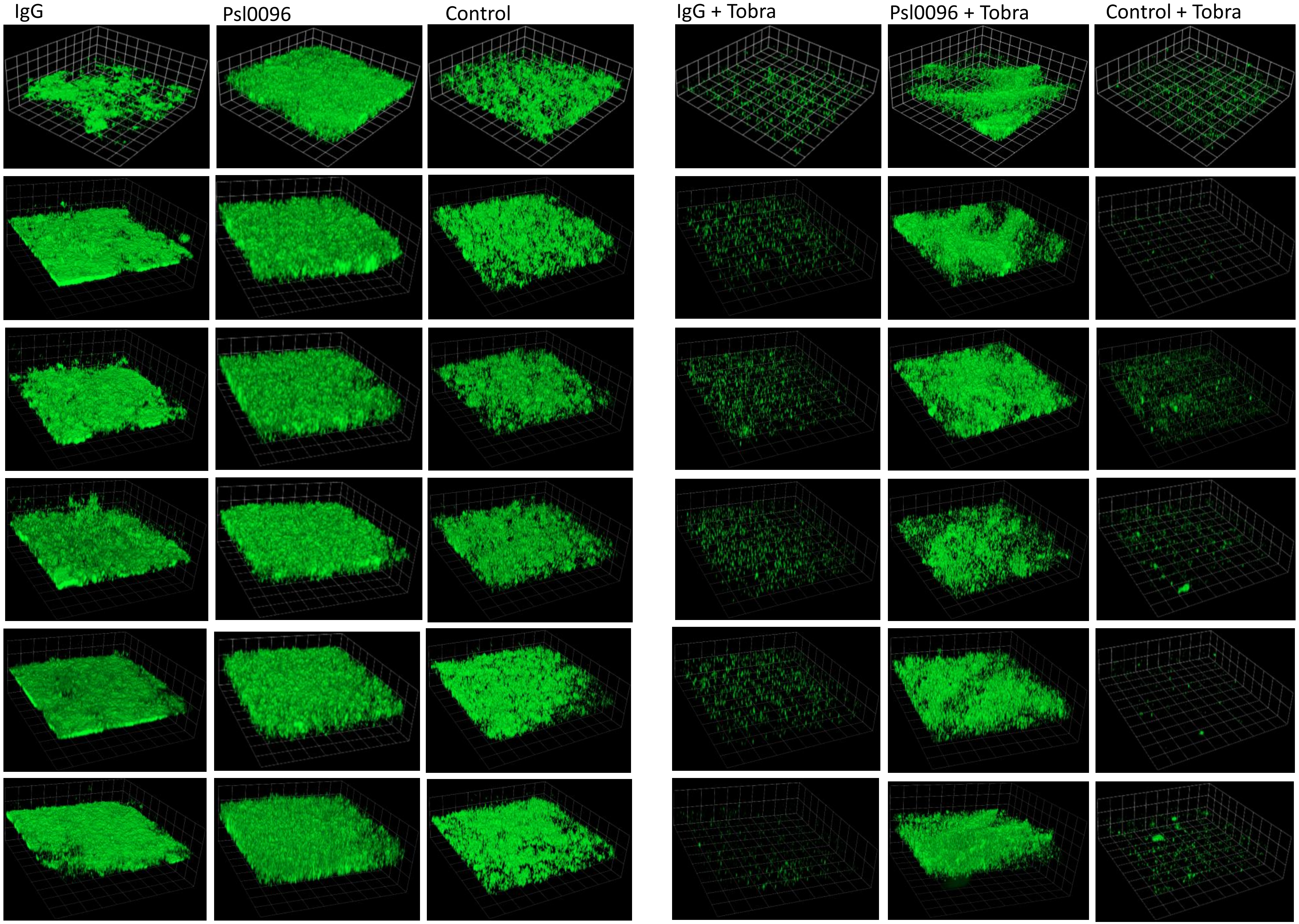

Pa551 (Persistent) Trial2

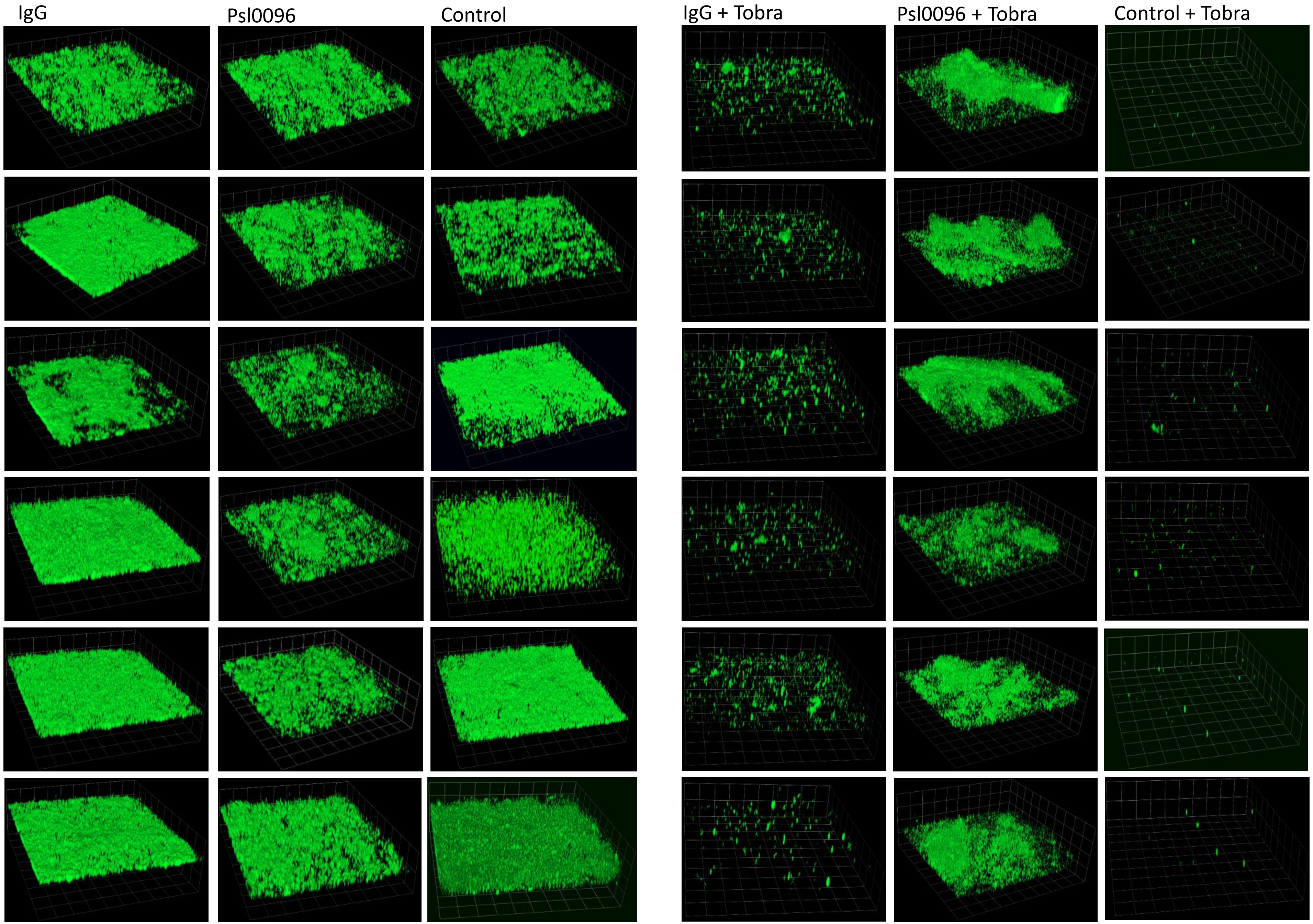

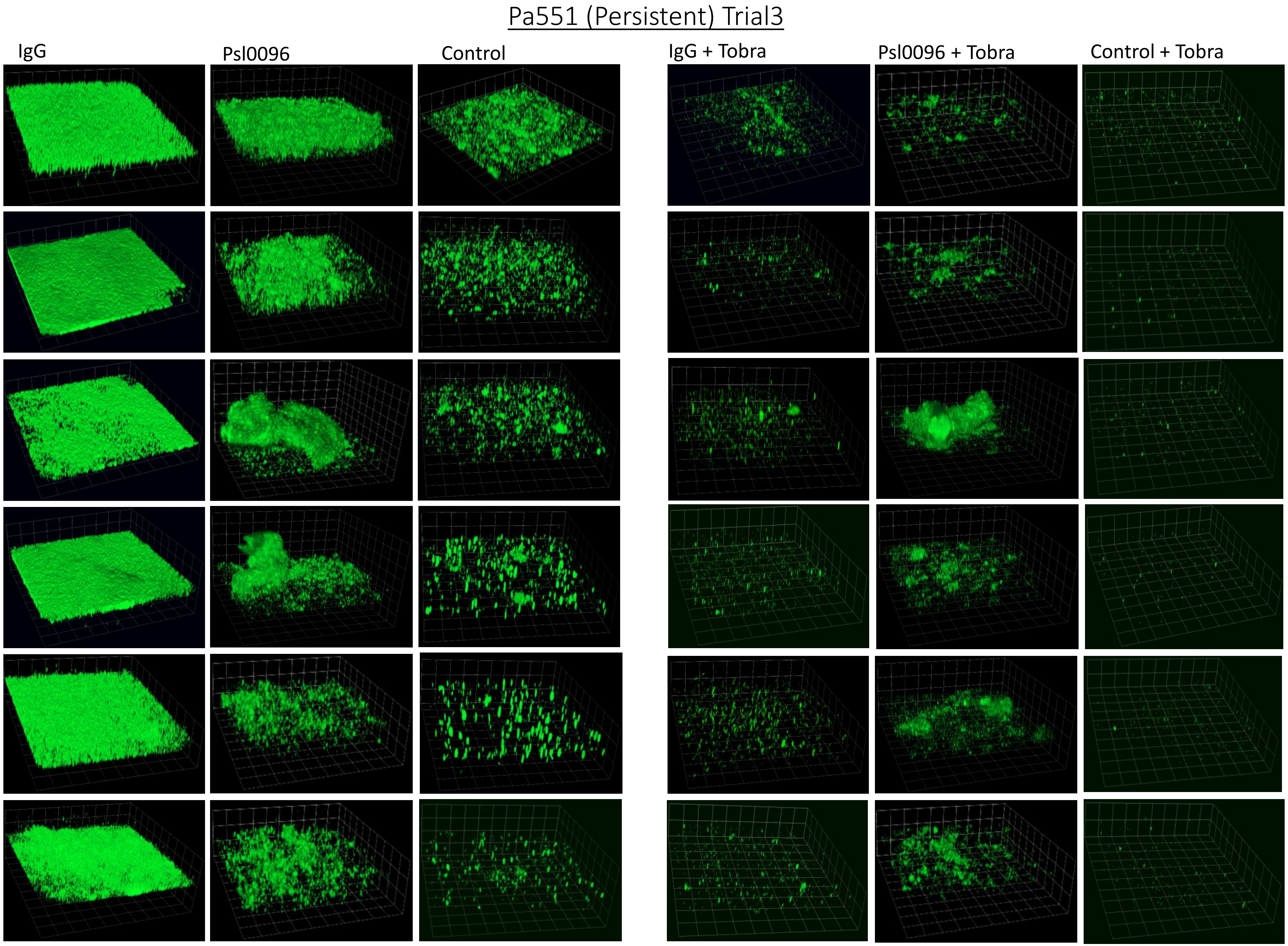
Pa551 (Persistent) Trial3
IgG
Psl0096
Control
IgG + Tobra
Psl0096 + Tobra
Control + Tobra

Pa549 (Eradicated) Trial1

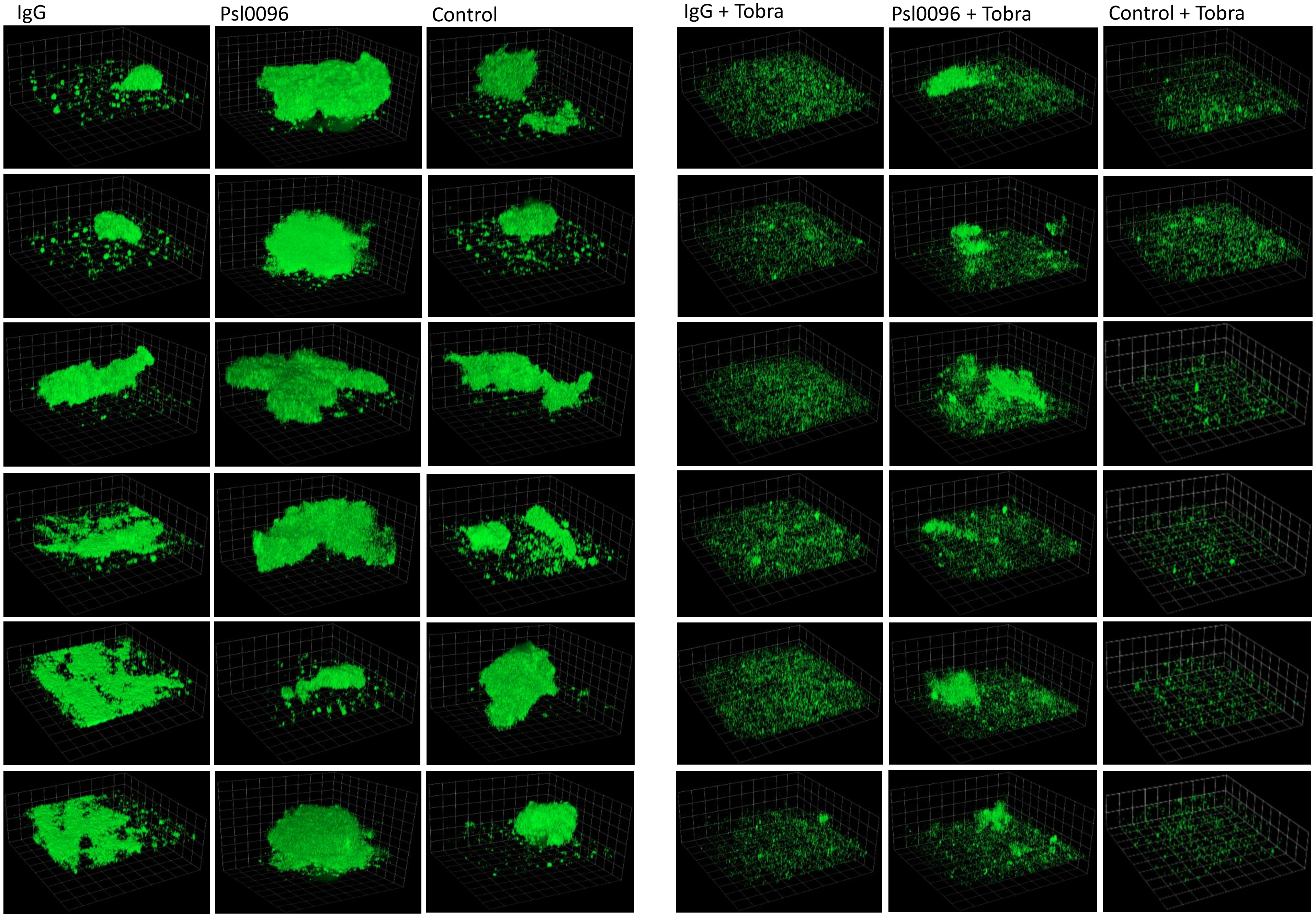

Pa549 (Eradicated) Trial2

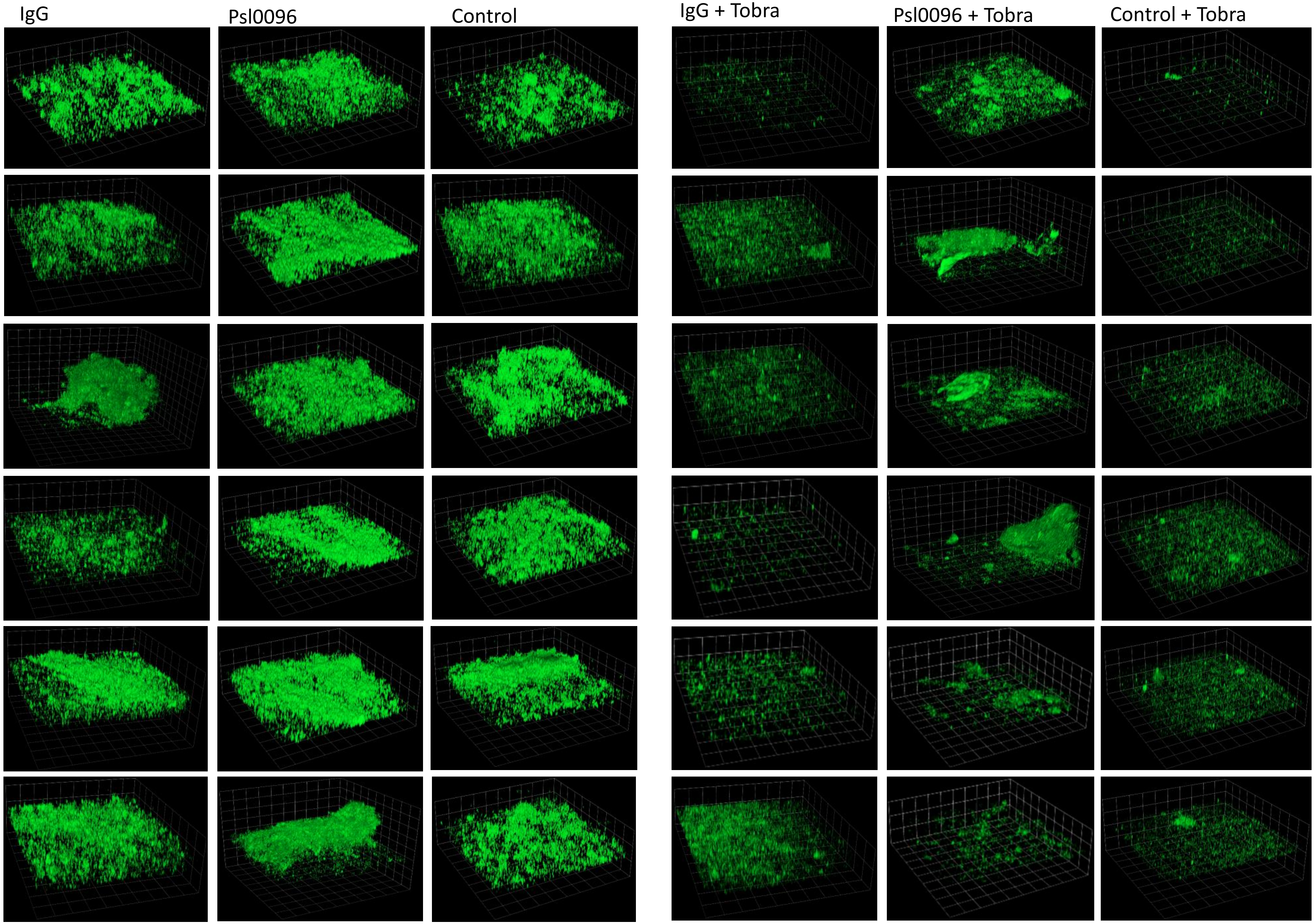

Pa549 (Eradicated) Trial3

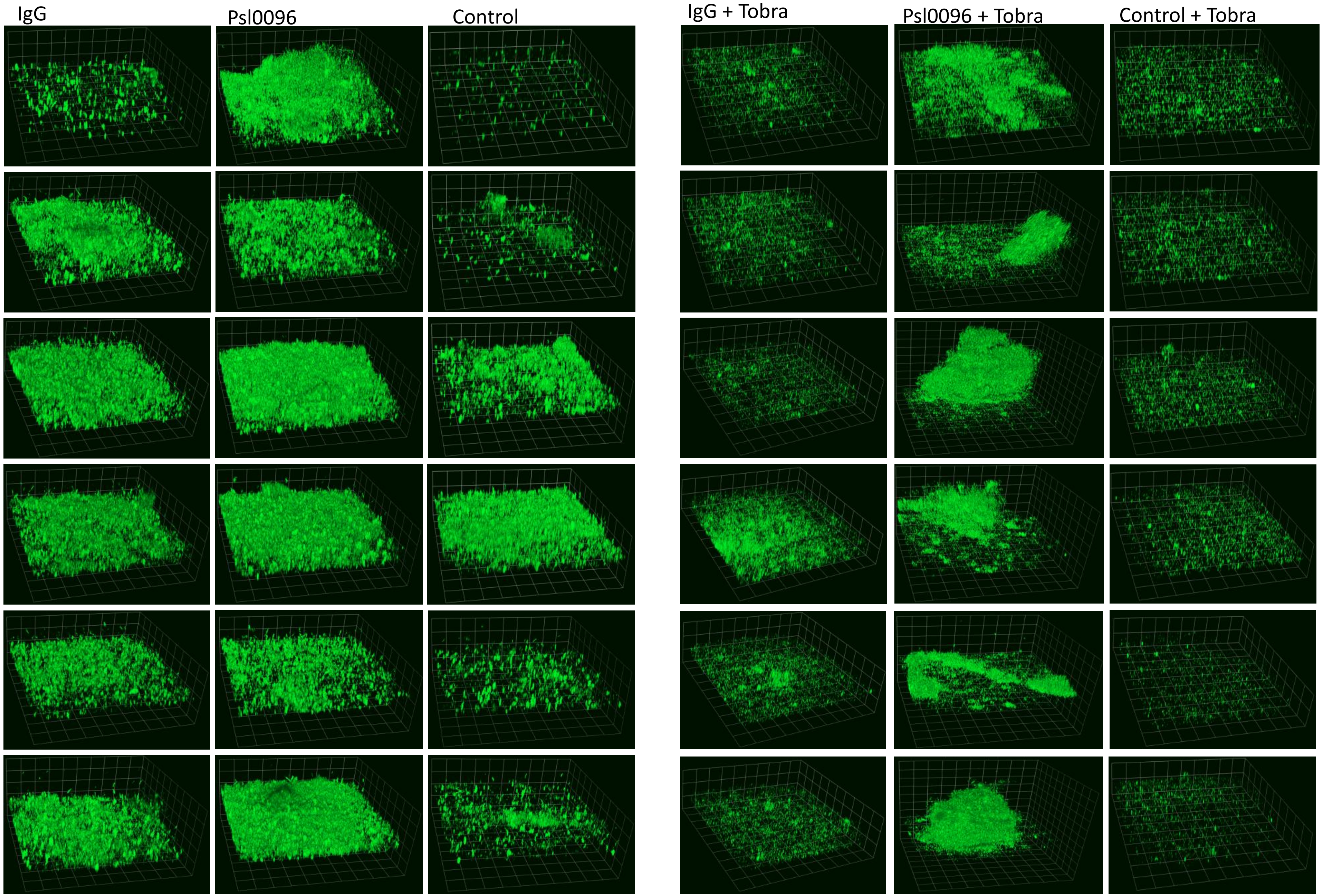

Pa404 (Eradicated) Trial1

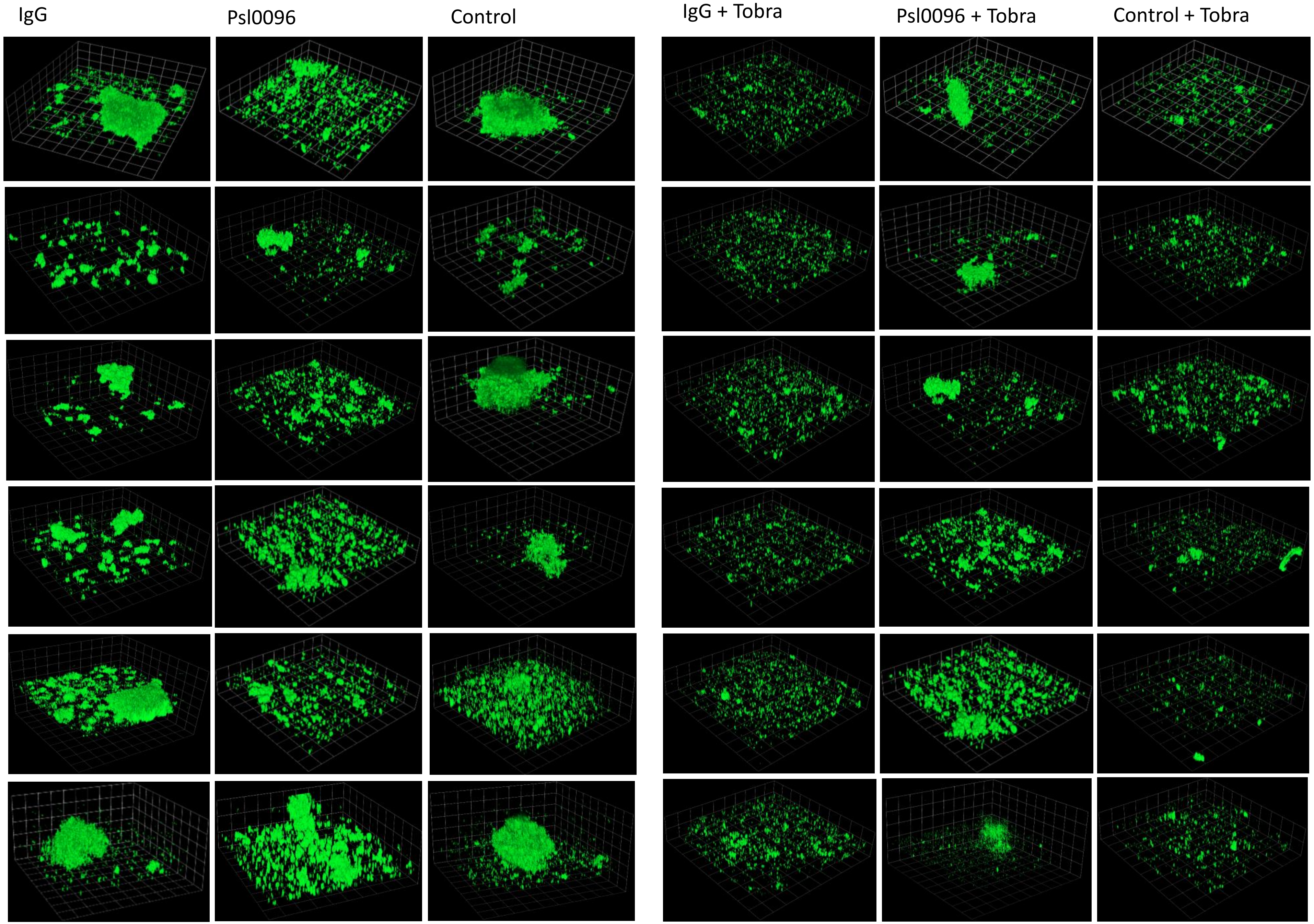

Pa404 (Eradicated) Trial2

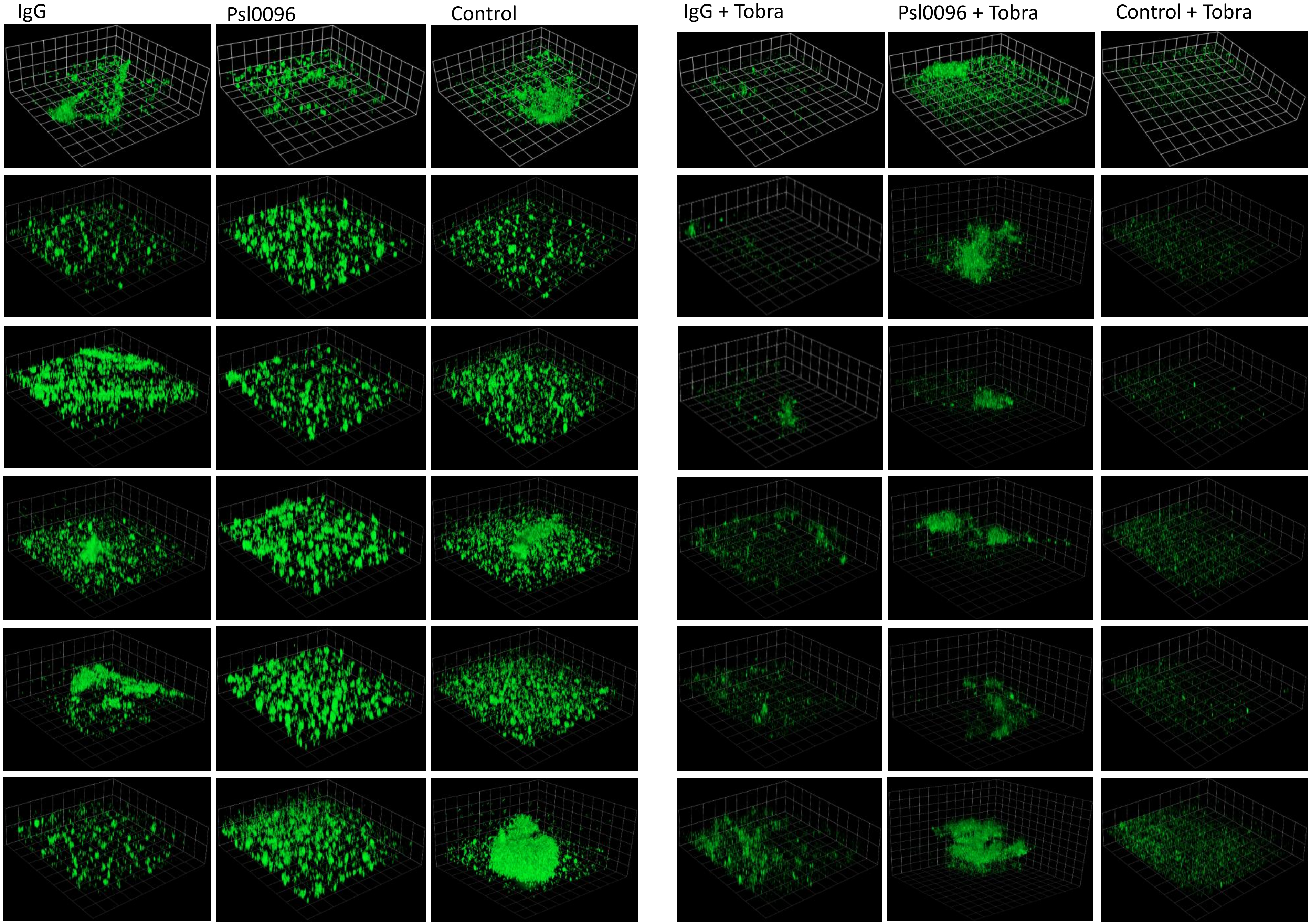

Pa404 (Eradicated) Trial3

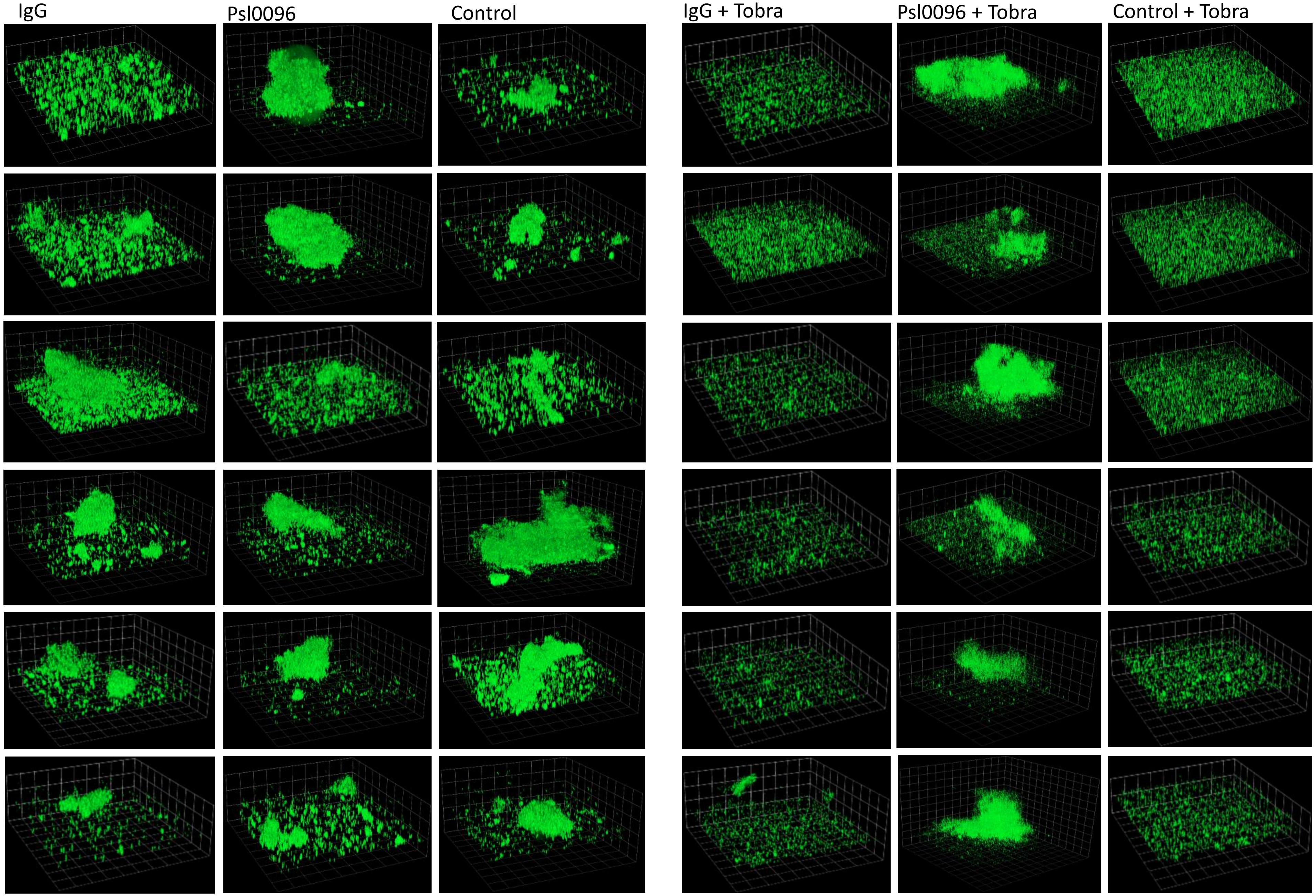

Pa288 (Eradicated) Trial1

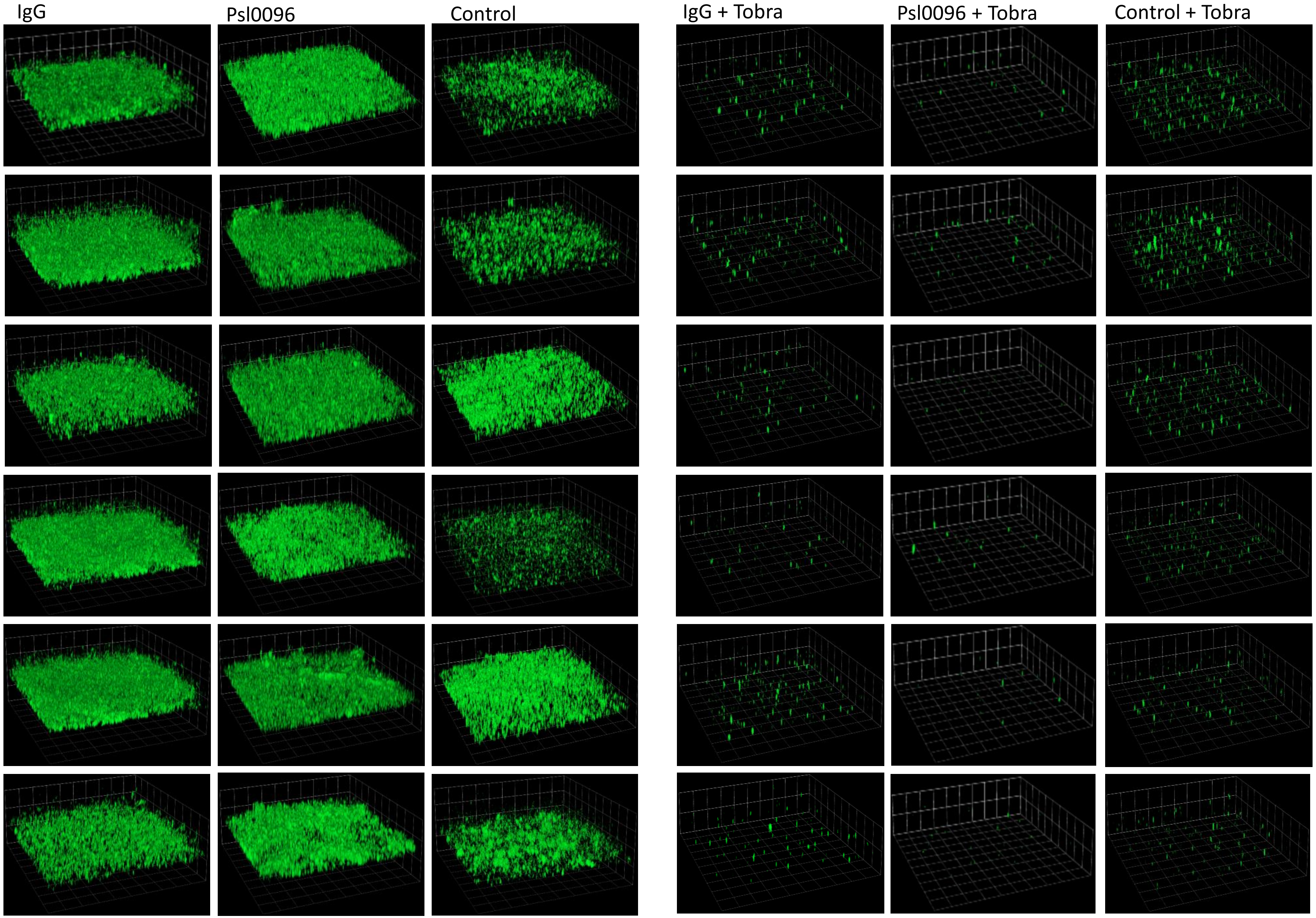

Pa288 (Eradicated) Trial2

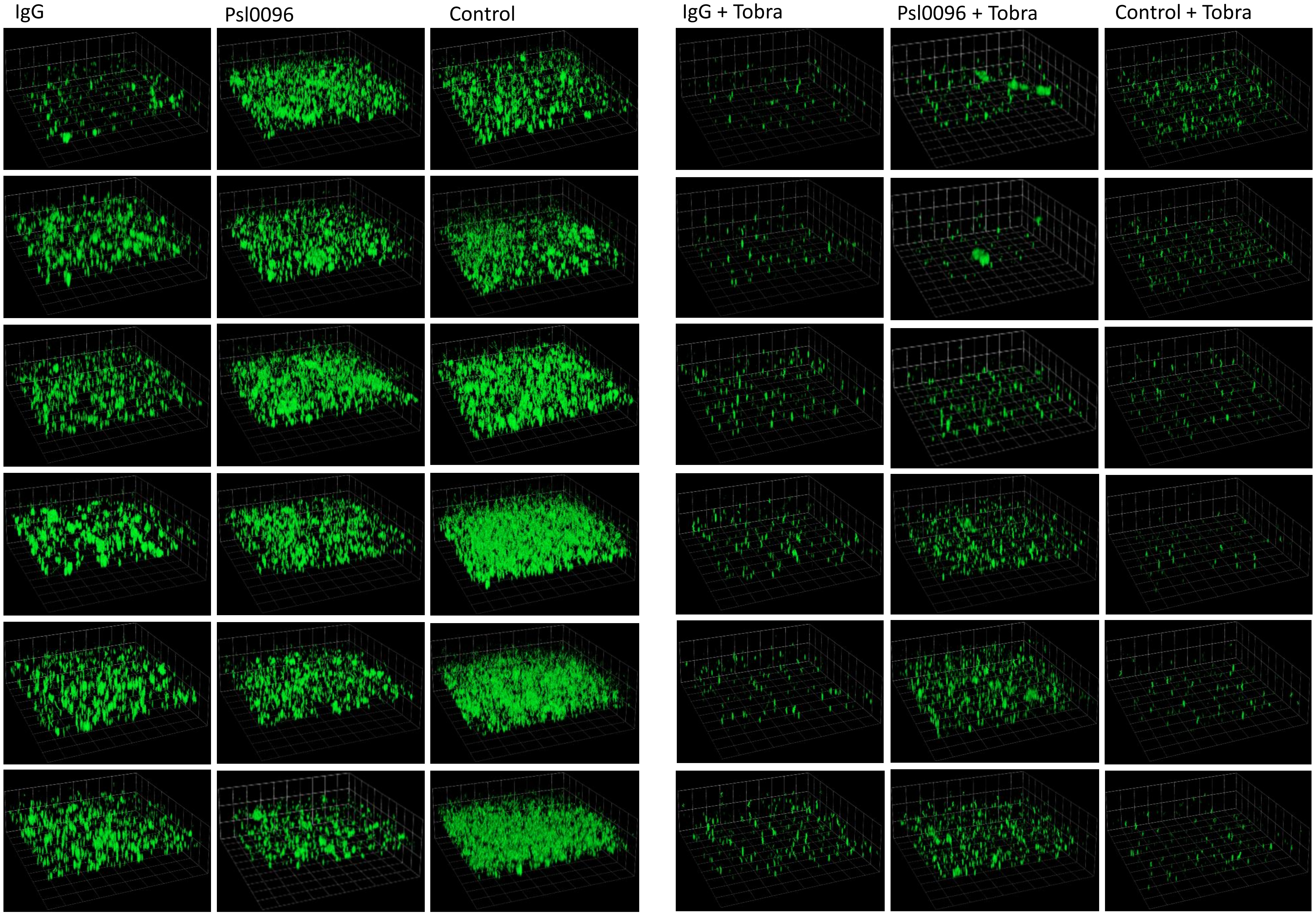

Pa288 (Eradicated) Trial3

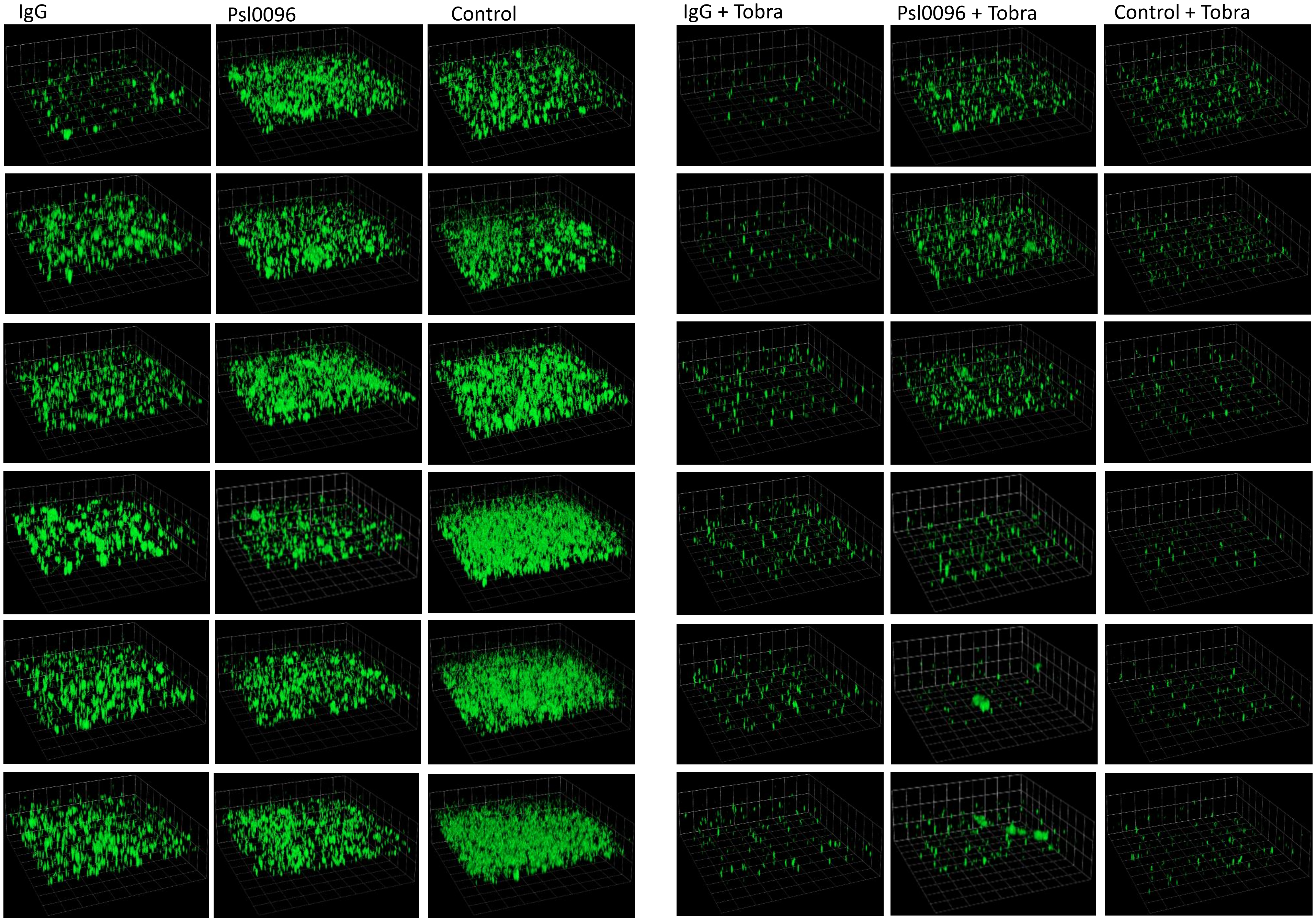

Pa325 (Eradicated) Trial1

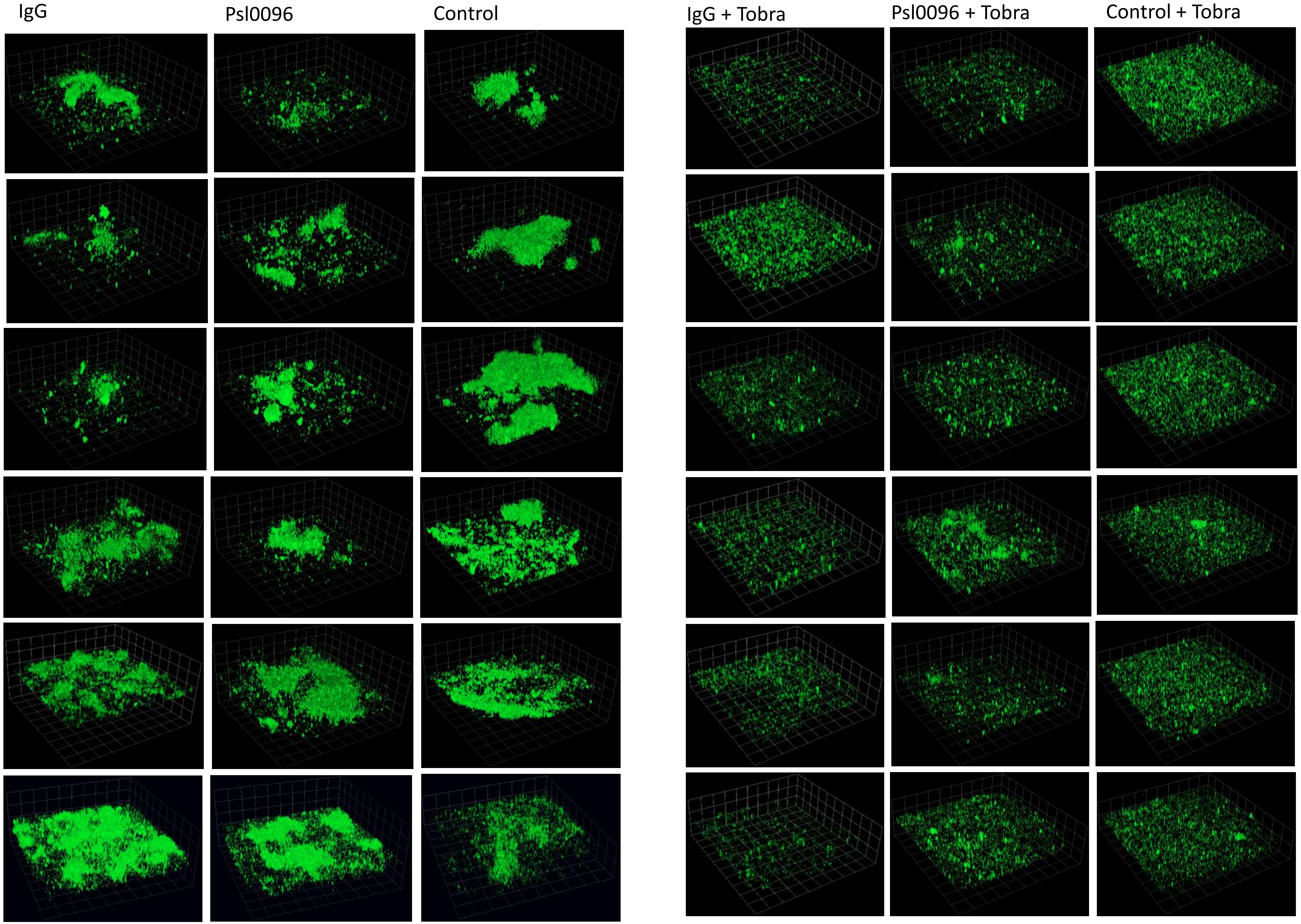

Pa325 (Eradicated) Trial2

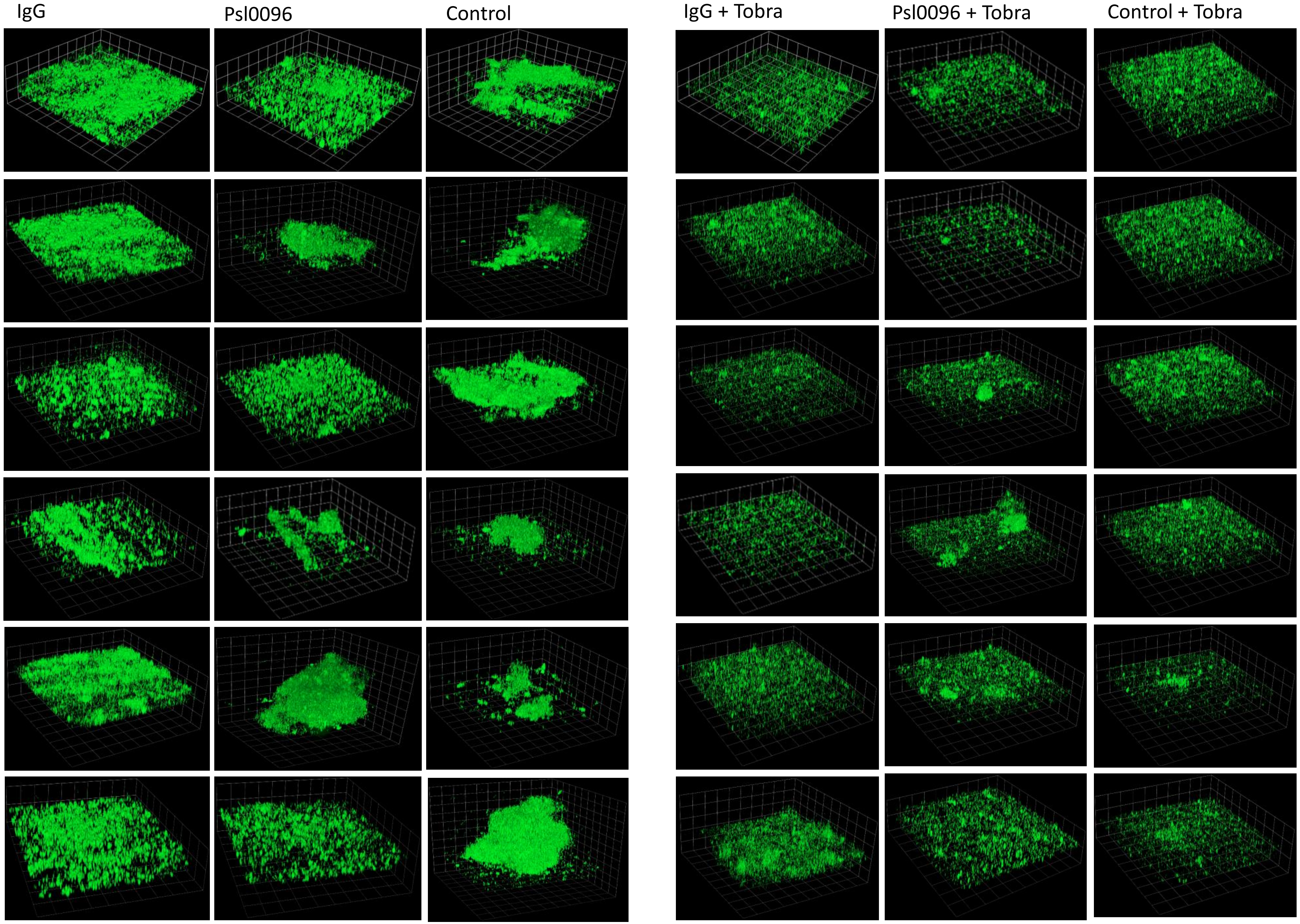

Pa325 (Eradicated) Trial3

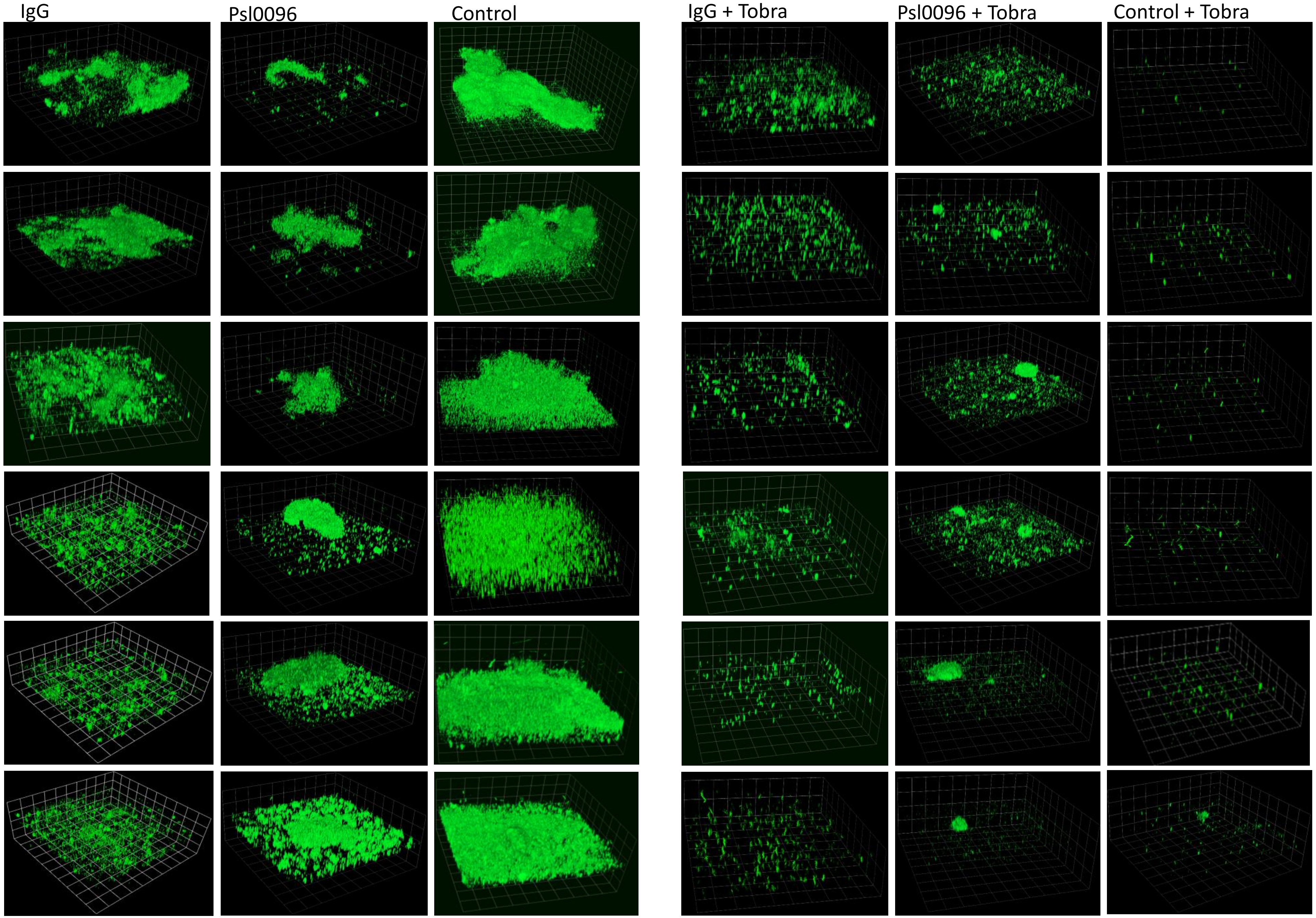

Pa263 (Eradicated) Trial1

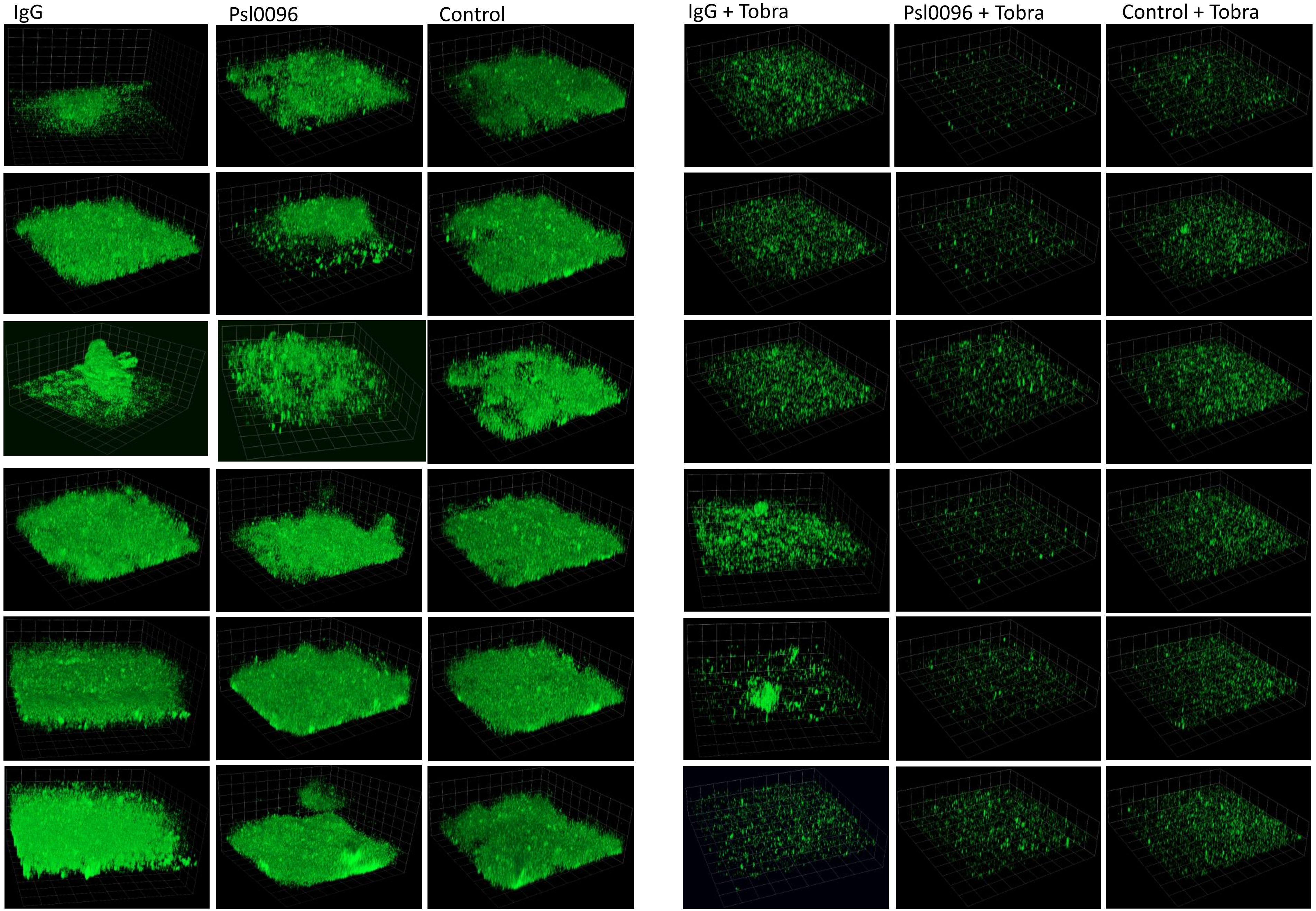

Pa263 (Eradicated) Trial2

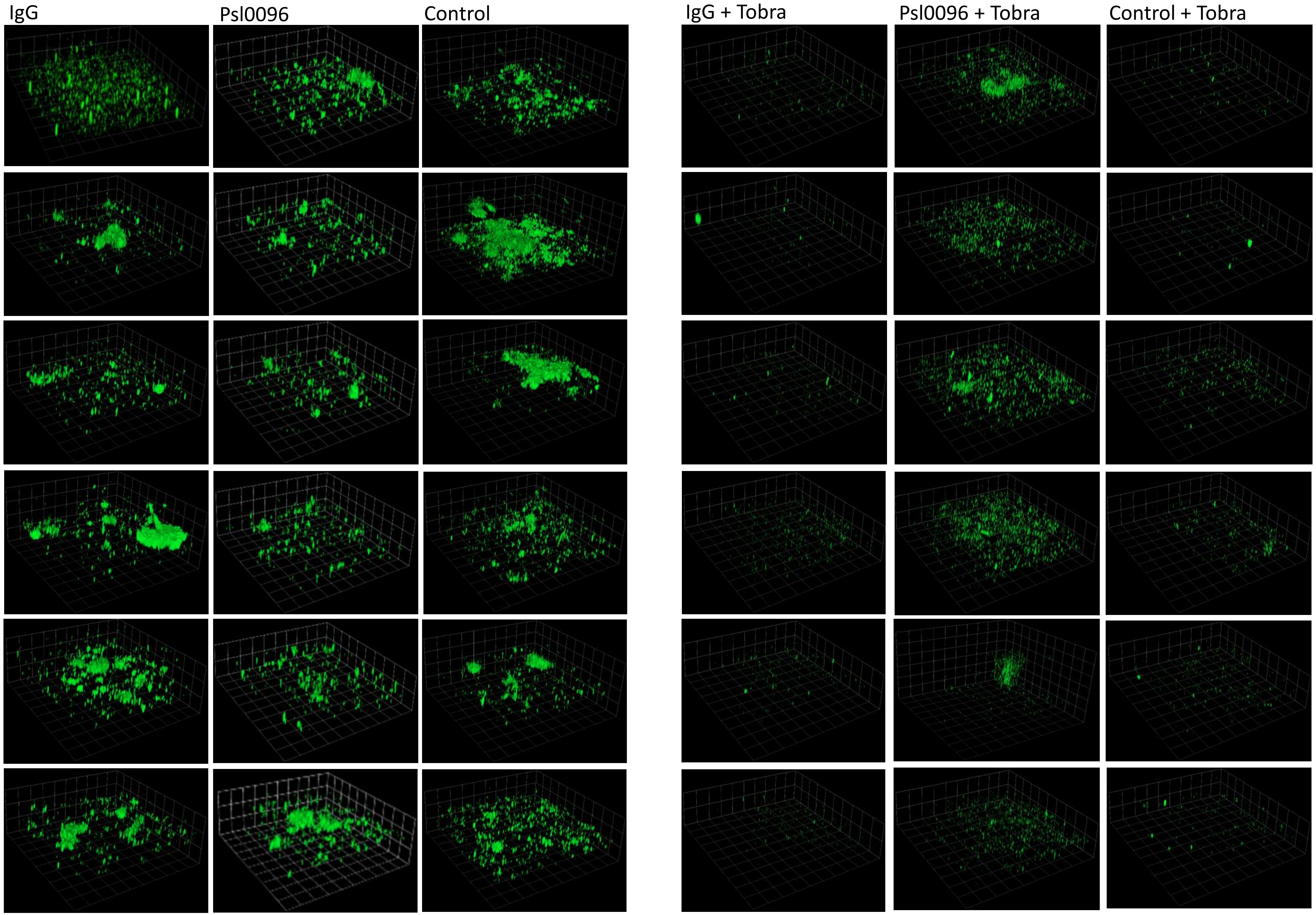

Pa263 (Eradicated) Trial3

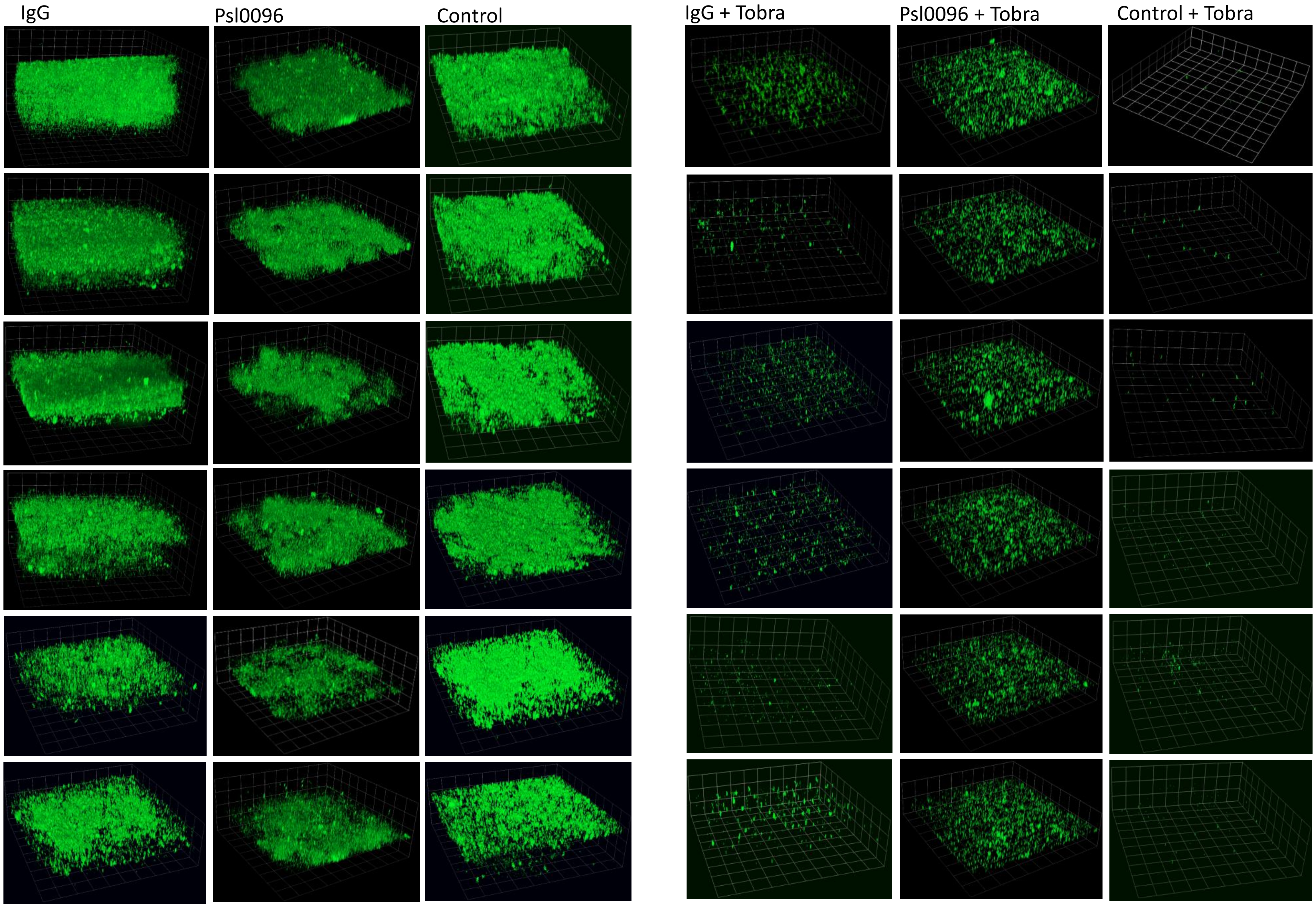

Pa50 (Eradicated) Trial1

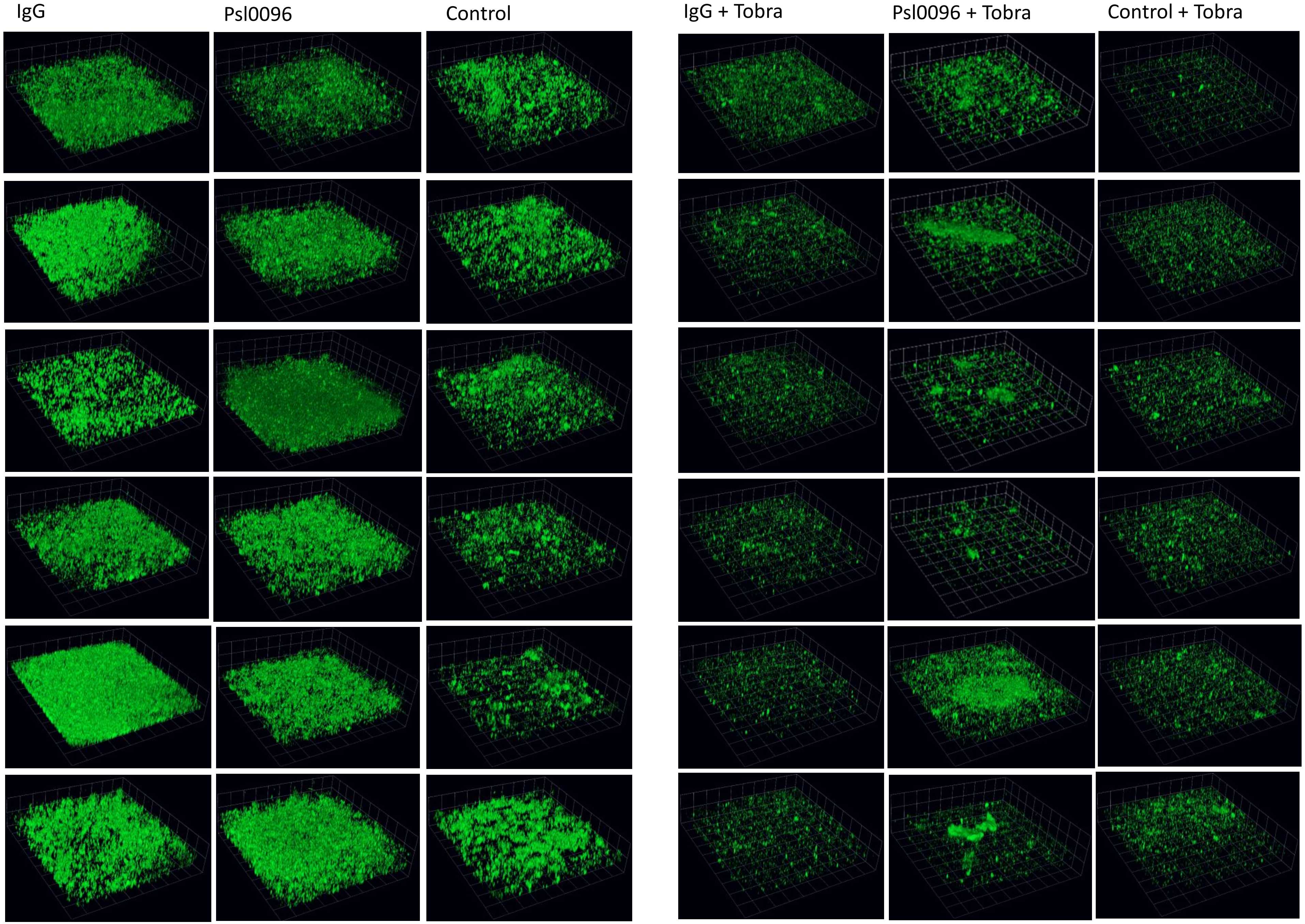

Pa50 (Eradicated) Trial2

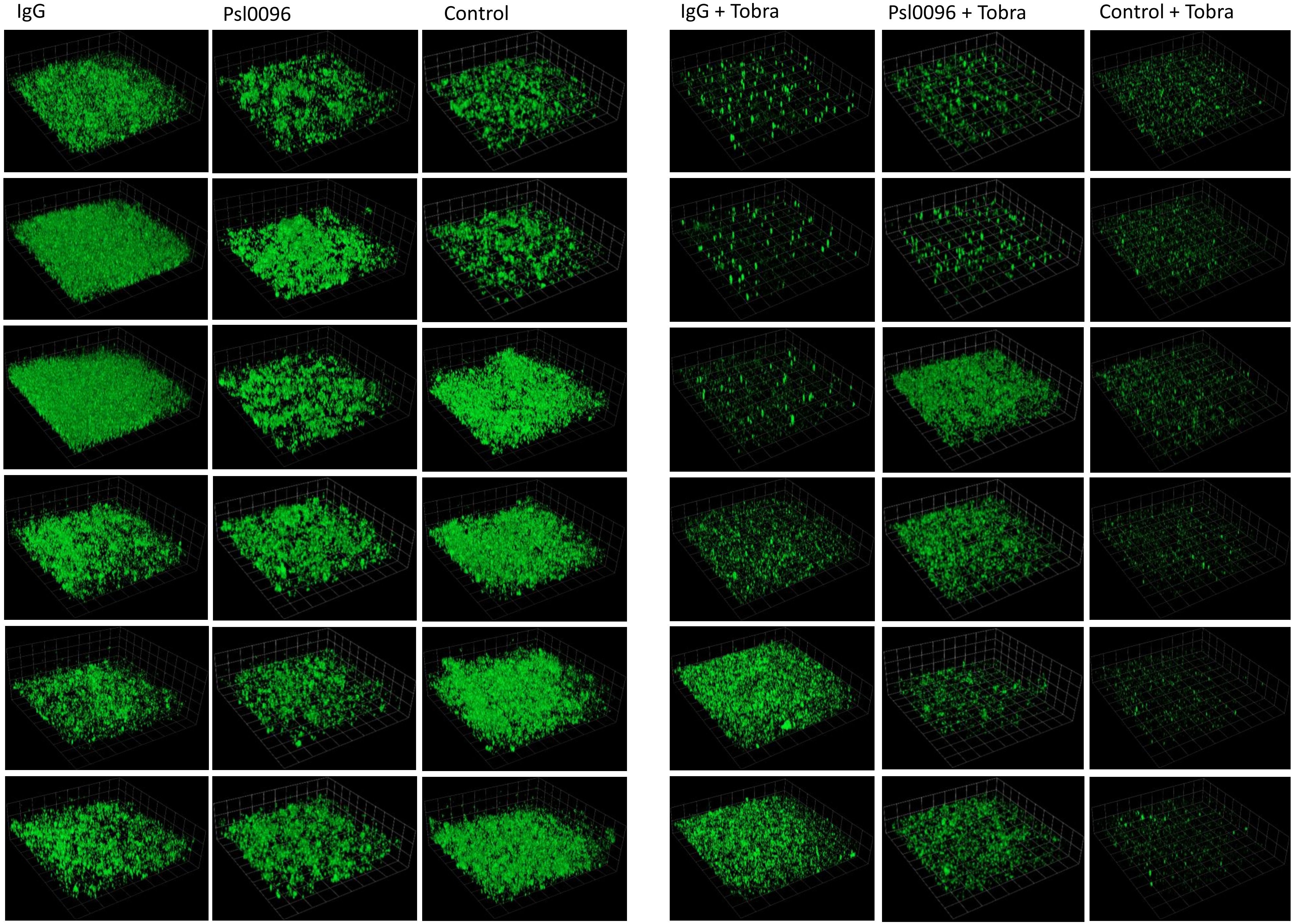

Pa50 (Eradicated) Trial3

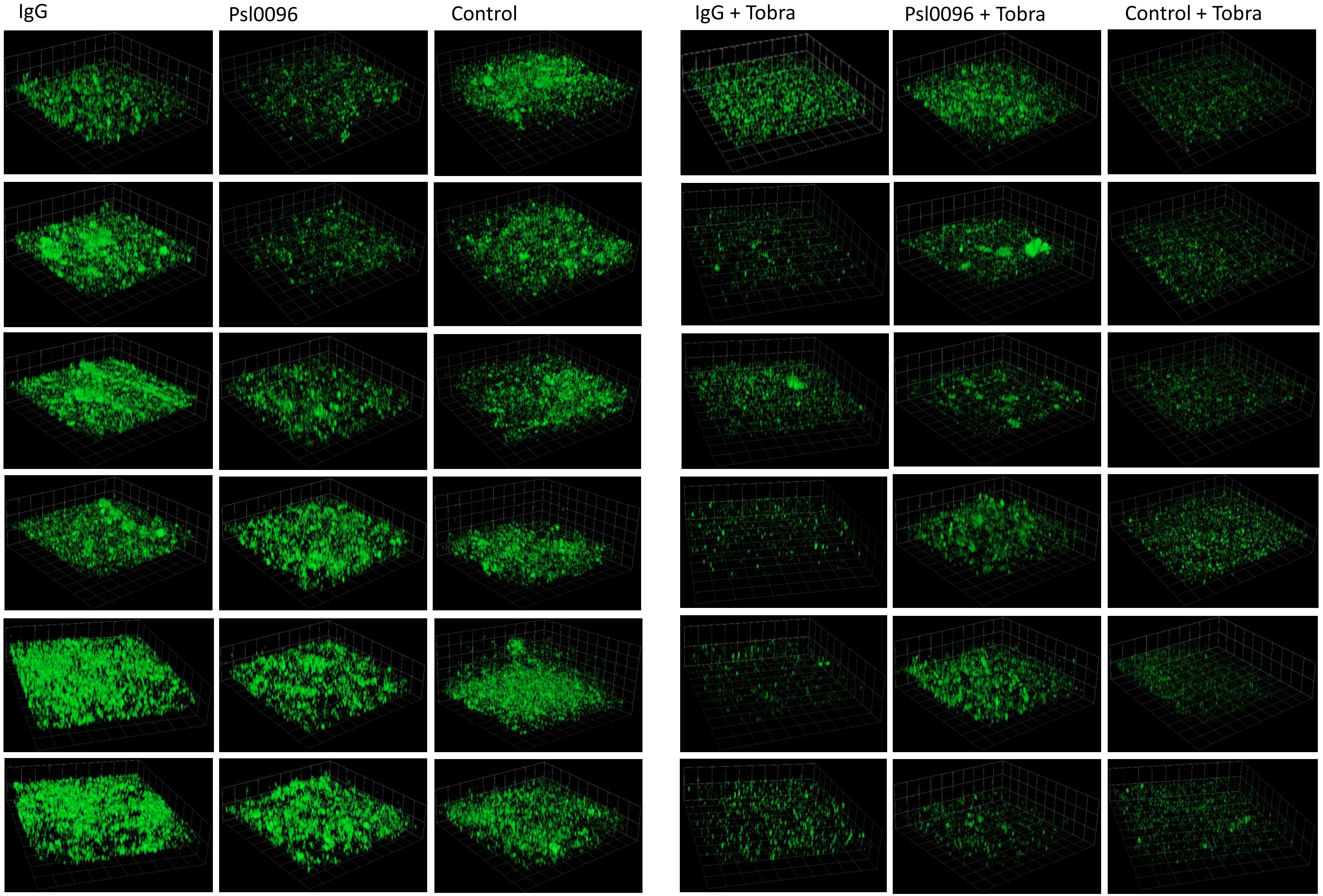

Pa558 (Eradicated) Trial1

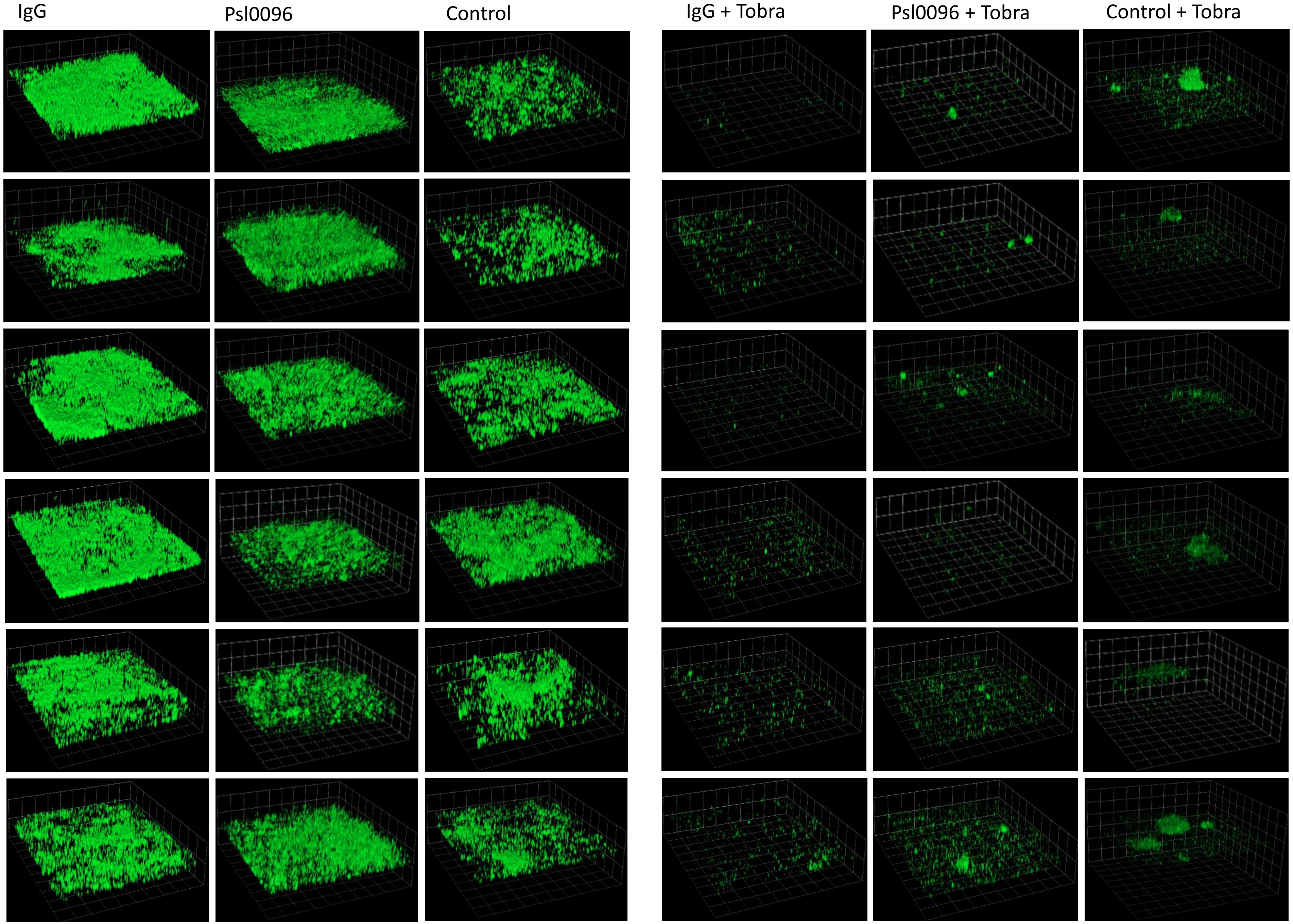

Pa558 (Eradicated) Trial2

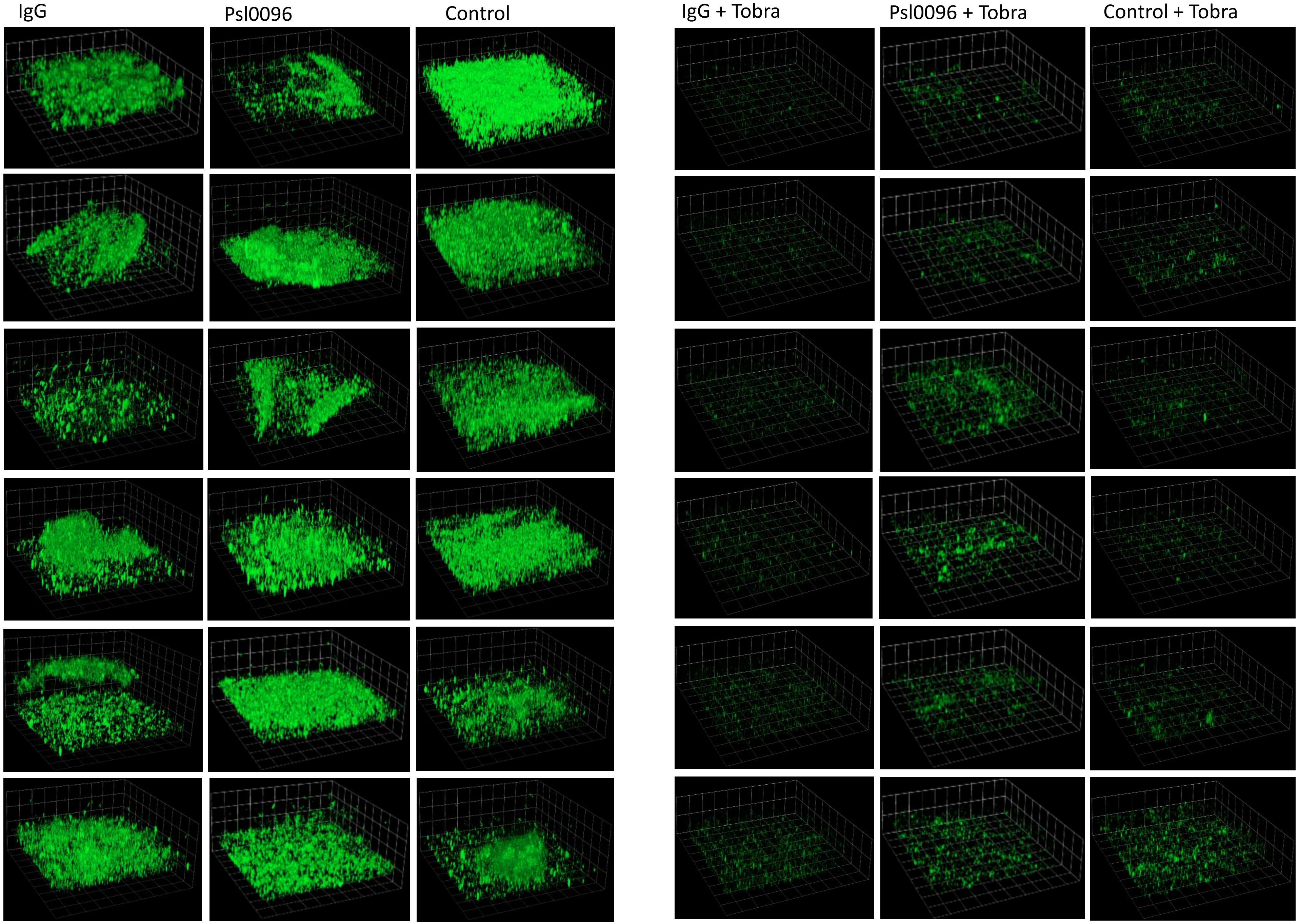

Pa558 (Eradicated) Trial3

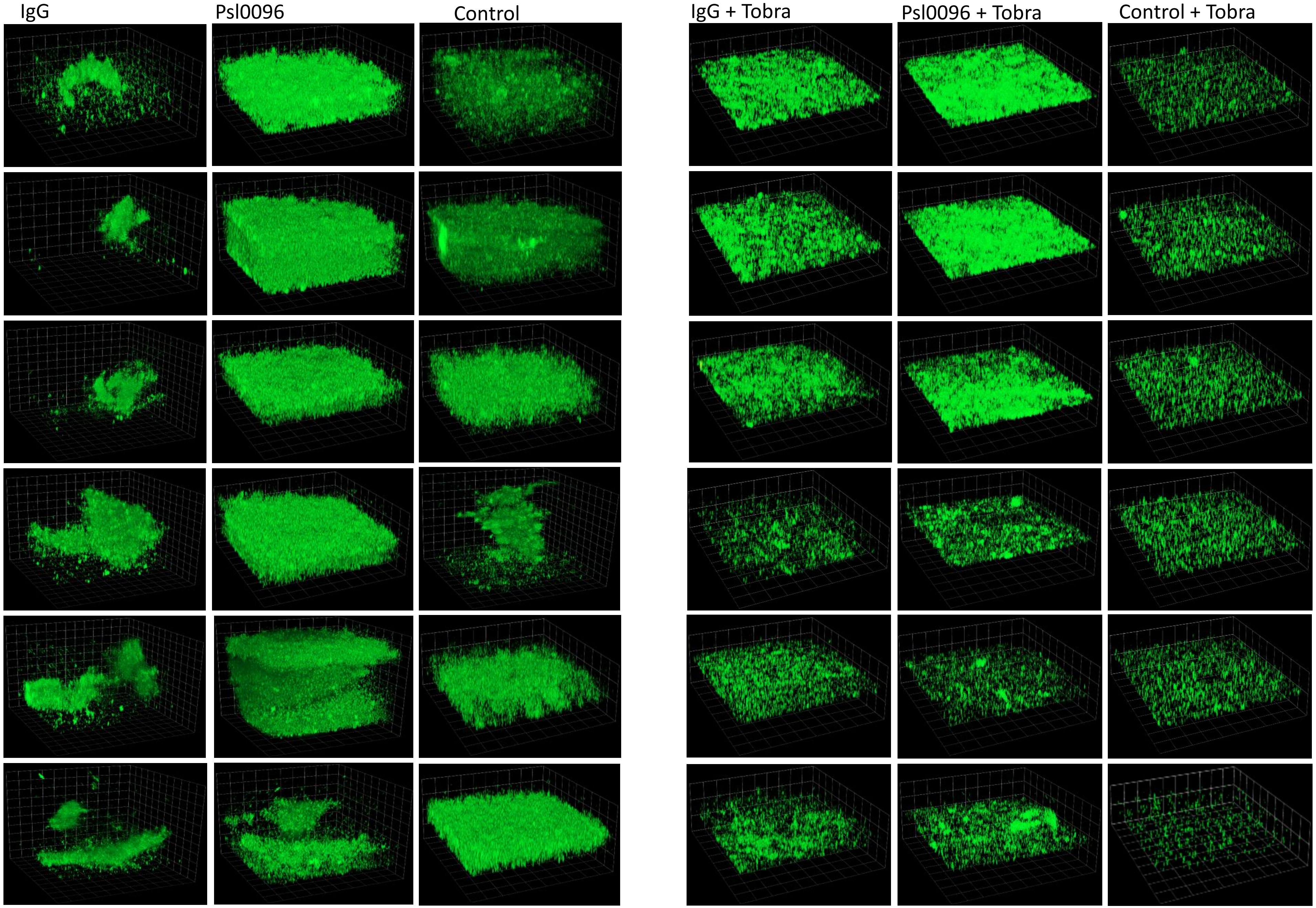

Supplement: Supplementary file 1 — Supplemental Materials [file 41522_2021_234_MOESM1_ESM.pdf]
